# Identification and functional characterization of two CXCL17 paralogs from zebrafish

**DOI:** 10.1042/BCJ20253418

**Published:** 2026-01-22

**Authors:** Jie Yu, Wen-Feng Hu, Juan-Juan Wang, Ya-Li Liu, Zeng-Guang Xu, Zhan-Yun Guo

**Affiliations:** 1 Research Center for Translational Medicine at East Hospital, School of Life Sciences and Technology, Tongji University, Shanghai, China

**Keywords:** activation, binding, chemotaxis, CXCL17, GPR25, zebrafish

## Abstract

C-X-C motif chemokine ligand 17 (CXCL17) and its receptor G protein-coupled receptor 25 (GPR25) have been identified as a significant pair in regulating immunity, but CXCL17 orthologs have not yet been identified from non-mammalian vertebrates. This study aimed to identify and characterize non-mammalian CXCL17 orthologs based on key features of mammalian CXCL17s, such as a C-terminal Xaa-Pro-Yaa motif, a signal peptide, and six cysteine residues. Two possible CXCL17 paralogs were identified from zebrafish (*Danio rerio*): Dr-CXCL17 (encoded by *zgc:158701*) and Dr-CXCL17-like (encoded by *si:dkey-112a7.5*). Both are previously uncharacterized proteins with unknown functions because they lack overall sequence similarity to known mammalian CXCL17s. For functional characterization, recombinant Dr-CXCL17 and Dr-CXCL17-like were prepared via overexpression in *Escherichia coli* and subsequent *in vitro* refolding, and their activity was tested using NanoLuc Binary Technology (NanoBiT)-based β-arrestin recruitment assays, NanoBiT-based ligand‒ receptor binding assays, and chemotaxis assays. The results showed that both Dr-CXCL17 and Dr-CXCL17-like tightly bound to and efficiently activated zebrafish GPR25 (Dr-GPR25) and induced chemotactic movement in transfected human embryonic kidney (HEK) 293T cells expressing the receptor. Deletion of three C-terminal residues in both paralogs almost eliminated their binding, activation, and chemotactic effects, which suggests that this fragment is crucial for their function. Homologs of Dr-CXCL17 or Dr-CXCL17-like were retrieved from several other ray-finned fish species, indicating that two CXCL17 paralogs are present in certain fish species and function as endogenous agonists for the fish GPR25 receptor. The identification of fish CXCL17 orthologs suggests that the CXCL17‒GPR25 pair likely originated in ancient fishes and was conserved across vertebrate lineages. This work represents the first identification of CXCL17 orthologs in non-mammalian vertebrates, paving the way for future functional studies of this ligand‒receptor pair.

## Introduction

C-X-C motif chemokine ligand 17 (CXCL17) functions as a chemoattractant for certain leukocytes, such as T cells, monocytes, macrophages, and dendritic cells [1–14]. It is also implicated in tumor development, likely through its regulation of tumor immunity [15–21]. However, the identity of its receptor remains controversial. The orphan G protein-coupled receptor 35 (GPR35) was first reported as its receptor in 2015 [22], but later studies did not support this pairing [23–25]. In recent years, both the chemokine receptor CXCR4 and the orphan MAS-related receptor MRGPRX2 were reported as its receptors [25,26]. Most recently, Ocón’s group and our group independently identified the orphan G protein-coupled receptor 25 (GPR25) as its receptor [27,28]. As a rarely studied A-class G protein-coupled receptor (GPCR), GPR25 is primarily expressed in specific immune cells, such as T cells, plasma cells, and B cells, according to data from the Human Protein Atlas (https://www.proteinatlas.org). The fact that these GPR25-expressing leukocytes can be attracted by mucosal tissue-expressed CXCL17 [27] suggests that the CXCL17–GPR25 pair plays an important role in immune regulation.

GPR25 orthologs are broadly distributed from fishes to mammals, but CXCL17 orthologs have not been identified in non-mammalian vertebrates so far. Consequently, the endogenous agonists of non-mammalian GPR25s remain unknown. A previous study reported that apelin and apela, two peptide hormones highly conserved among vertebrates, act as weak agonists for certain non-mammalian GPR25s [29]. Our recent work demonstrated that human CXCL17 exhibits markedly higher activity than apelin or apela toward fish GPR25s from both the ray-finned zebrafish (*Danio rerio*) and the lobe-finned coelacanth (*Latimeria chalumnae*) [30]. Based on these findings, we hypothesized that CXCL17 orthologs may indeed exist in non-mammalian vertebrates but remain unrecognized due to the absence of overall sequence similarity with known mammalian CXCL17s.

In this study, we identified and functionally characterized two zebrafish CXCL17 paralogs, designated as Dr-CXCL17 and Dr-CXCL17-like, based on key sequence features shared with mammalian CXCL17s. Both proteins were previously uncharacterized and of unknown function, as their lack of overall amino acid sequence similarity with mammalian CXCL17s precluded their recognition as CXCL17 orthologs. Recombinant Dr-CXCL17 and Dr-CXCL17-like were shown to bind to and activate zebrafish GPR25 (Dr-GPR25) and to induce chemotactic migration of transfected human embryonic kidney (HEK) 293T cells via their conserved C-terminal fragment, indicating that they act as endogenous agonists of GPR25 in zebrafish. Database searches further identified fish homologs through BLAST analysis using Dr-CXCL17 or Dr-CXCL17-like, suggesting that these two paralogs are present in certain fish species. To the best of our knowledge, this is the first report of CXCL17 orthologs in non-mammalian vertebrates, providing a foundation for future functional studies of the CXCL17–GPR25 pair in these species.

## Results

### Identification of possible fish CXCL17s

Our recent studies have shown that three C-terminal residues are essential for human CXCL17 to activate human GPR25, as well as zebrafish and coelacanth GPR25s [28,30]. These residues are highly conserved among mammalian CXCL17s, particularly the penultimate proline (Pro) residue (Supplementary Figure S1 and Supplementary Table S1). We therefore hypothesized that, if present, fish CXCL17 orthologs would possess three analogous residues at their C-terminus. Based on this, we proposed a potential C-terminal motif for fish CXCL17s: Xaa-Pro-Yaa, where Xaa and Yaa are typically large aliphatic residues such as leucine (Leu), isoleucine (Ile), methionine (Met), or valine (Val). This motif served as a primary criterion for searching publicly available databases for candidate fish CXCL17s. In addition, we applied two supplementary criteria: (1) they should be secretory proteins, possessing an N-terminal signal peptide; and (2) their mature peptides should contain six cysteine (Cys) residues and be fewer than 200 amino acids in length.

Zebrafish (*Danio rerio*) is a widely used model organism whose genome has been fully sequenced, extensively analyzed, and well annotated. We retrieved all zebrafish secretory proteins with fewer than 200 amino acids from the UniProt database (https://www.uniprot.org/uniprotkb?query=Danio+rerio&facets=model_organism%3A7955%2Cproteins_with%3A49%2Clength%3A%5B1+TO+200%5D) and searched for potential CXCL17s among them using the proposed criteria. Among the 795 proteins obtained, two candidates were identified with UniProt IDs B0S594 and A0AB13AB19, respectively.

The genetic information for both zebrafish proteins is available in the NCBI gene database according to the reference genome (GRCz12tu) of the Tübingen strain zebrafish (Supplementary Table S2 and S3). B0S594 is encoded by the gene *zgc:158701* (Gene ID: 100151367) and located between *pafah1b3* and *ceacam1* on zebrafish chromosome 16 (Supplementary Figure S2). This gene is composed of four exons and three introns and produces a single mRNA (NM_001144821) that encodes a small secretory protein (NP_001138293) comprising 109 amino acids (Supplementary Figure S2). A0AB13AB19 is encoded by the gene *si:dkey-112a7.5* (Gene ID: 100536854) and situated between *ponzr5* and *ugt5g1* on zebrafish chromosome 7 (Supplementary Figure S3). This gene is also composed of four exons and three introns, but it yields two mRNA transcripts (NM_001386806 and XM_073906074), both of which encode the same protein (NP_001373735 or XP_073762175) containing 95 amino acids (Supplementary Figure S3). For clarity, in this study, we designated B0S594 as Dr-CXCL17 and A0AB13AB19 as Dr-CXCL17-like.

According to the RNA sequencing data shown in the zebrafish reference genome (GRCz12tu), the genes of Dr-CXCL17 and Dr-CXCL17-like are transcribed (Supplementary Figure S2,S3). To experimentally confirm their expression, we conducted reverse transcription and cDNA cloning (Supplementary Figure S2,S3). As analyzed by agarose gel electrophoresis, ~400 bp DNA fragments were successfully amplified for both genes (Supplementary Figure S4B). After ligation into a vector, two transformed *Escherichia coli* colonies were subjected to DNA sequencing: they were identical to the reference cDNA sequence of Dr-CXCL17 (NM_001144821) and Dr-CXCL17-like (NM_001386806), respectively. Similarly, cDNA of the zebrafish GPR25 (Dr-GPR25) was PCR amplified and sequenced (Supplementary Figure S4): one colony was identical to the reference sequence of Dr-GPR25 (XM_073916757), but the other colony contained six synonymous mutations. Thus, our results confirmed that Dr-CXCL17, Dr-CXCL17-like, and Dr-GPR25 are expressed in zebrafish, implying they are functional.

**Figure 1:**
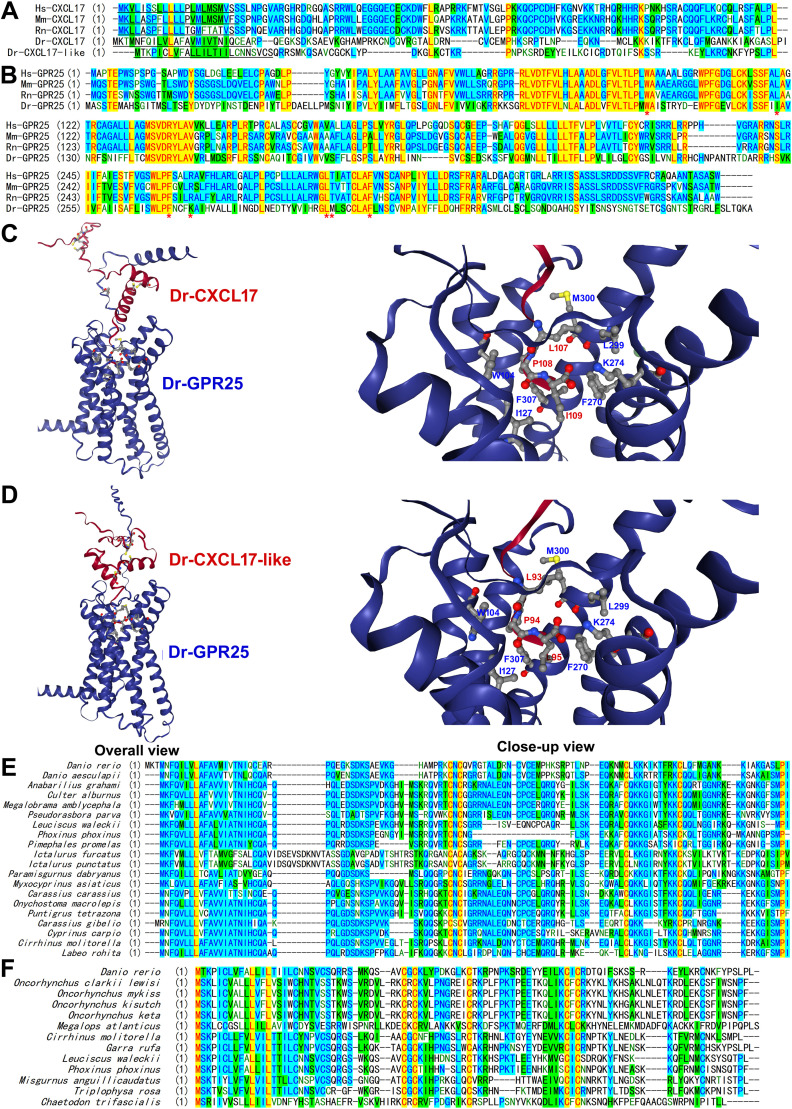
Identification of possible fish CXCL17s. (**A**) Amino acid sequence alignment of CXCL17s from human, mouse, rat, or zebrafish. These proteins were retrieved from NCBI database (Supplementary Table S1‒S3) and aligned via AlignX algorithm using the Vector NTI 11.5.1 software. Their signal peptide was predicted by SignalP-6.0 algorithm (https://services.healthtech.dtu.dk/services/SignalP-6.0/) and underlined. (**B**) Amino acid sequence alignment of GPR25s from human, mouse, rat, or zebrafish. Hs-GPR25 (NP_005289), Mm-GPR25 (NP_001094986), Rn-GPR25 (NP_001385523), and Dr-GPR25 (XP_073772858) were aligned via AlignX algorithm using the Vector NTI 11.5.1 software. The Dr-GPR25 residues involving ligand-binding are indicated by red asterisks. (**C,D**) AlphaFold3-predicted binding of the mature Dr-CXCL17 (**C**) and Dr-CXCL17-like (**D**) with Dr-GPR25. The binding structures were predicted via the online AlphaFold3 server (https://alphafoldserver.com) and viewed by an online server (https://nglviewer.org/ngl). The six Cys residues and three C-terminal residues of Dr-CXCL17 and Dr-CXCL17-like are shown as balls-and-sticks. The residues of Dr-GPR25 involving ligand-binding are shown as sticks-and-balls and indicated in panel B by red asterisks. The predicted possible interactions are summarized in Supplementary Table S4 **E,F**) Amino acid sequence alignment of the fish homologs of Dr-CXCL17 (**E**) or Dr-CXCL17-like (**F**). These homologs were retrieved from the NCBI database via blast with Dr-CXCL17 or Dr-CXCL17-like (https://blast.ncbi.nlm.nih.gov), and their information is shown in Supplementary Table S2 and S3. Some fish CXCL17 homologs have two variants (Supplementary Table S2) due to alternative splicing and only the longer ones were aligned in panel D. The sequence alignment was conducted via AlignX algorithm using the Vector NTI 11.5.1 software.

As shown in Figure 1A, neither Dr-CXCL17 nor Dr-CXCL17-like exhibits significant overall sequence similarity to human CXCL17 (Hs-CXCL17), mouse CXCL17 (Mm-CXCL17), or rat CXCL17 (Rn-CXCL17) and thus cannot be identified through sequence BLAST searches using mammalian CXCL17s as queries. Consequently, both proteins were previously uncharacterized and of unknown function. In contrast, Dr-GPR25 shares high sequence similarity with human GPR25 (Hs-GPR25), mouse GPR25 (Mm-GPR25), and rat GPR25 (Rn-GPR25) (Figure 1B), despite the evolutionary divergence of mammals and fishes approximately 400 million years ago.

The full-length Dr-CXCL17 contains a predicted N-terminal signal peptide of 25 residues and a mature peptide of 84 amino acids, whereas the full-length Dr-CXCL17-like has a predicted N-terminal signal peptide of 25 residues and a mature peptide of 70 amino acids (Figure 1A). Both proteins contain six cysteine (Cys) residues in the mature peptide; however, their Cys arrangements differ: CXC-CXC-C-C in Dr-CXCL17 and CXC-C-CXC-C in Dr-CXCL17-like (Figure 1A). The Cys pattern of Dr-CXCL17 is identical to that of mammalian CXCL17s, while the arrangement in Dr-CXCL17-like is distinct. The mature forms of both proteins are basic, with predicted isoelectric point (pI) values of 10.5 and 10.0, respectively. This basic nature may underlie their chemoattractant activity, as it could facilitate binding to negatively charged cell surface glycosaminoglycans after secretion, thereby forming local concentration gradients to attract immune cells.

According to AlphaFold2 predictions (AF-B0S594-F1-model_v4), Dr-CXCL17 adopts a flexible structure with three disulfide bonds (C47–C62, C49–C60, and C80–C91), but the overall prediction confidence is low. AlphaFold3 modeling further predicts that Dr-CXCL17 binds to Dr-GPR25 with an ipTM value of approximately 0.6, with the ligand’s C-terminal fragment inserted into the orthosteric ligand-binding pocket of the receptor (Figure 1C). The three conserved C-terminal residues (I109, P108, and L107) of the ligand form extensive interactions with counterpart residues of Dr-GPR25 (Figure 1C and Supplementary Table S4). For example, the negatively charged C-terminal carboxyl moiety of Dr-CXCL17 forms electrostatic interactions with the positively charged ε-amine moiety of the receptor’s K274 residue; the penultimate P108 of the ligand forms hydrophobic interactions with the aromatic W104 of the receptor (Figure 1C and Supplementary Table S4).

AlphaFold3 predictions indicate that the mature Dr-CXCL17-like (residues 26–95) adopts a compact globular structure with three disulfide linkages (C36–C66, C38–C48, and C68–C86) (Figure 1D). AlphaFold3 modeling also predicts binding of Dr-CXCL17-like to Dr-GPR25 with an ipTM value of approximately 0.6, in which the ligand’s C-terminal fragment inserts into the orthosteric ligand-binding pocket of the receptor (Figure 1D). The three conserved C-terminal residues (L95, P94, and L93) of Dr-CXCL17-like form extensive interactions with counterpart residues of the receptor (Figure 1D and Supplementary Table S4). According to the AlphaFold3 predictions, Dr-CXCL17 and Dr-CXCL17-like have similar interaction patterns with receptor Dr-GPR25 (Supplementary Table S4).

Amino acid sequence BLAST searches using Dr-CXCL17 identified several fish homologs in publicly available databases (Figure 1E and Supplementary Table S2). These homologs typically possess an N-terminal signal peptide for secretion, a mature peptide containing six cysteine residues arranged in the CXC-CXC-C-C pattern, and three highly conserved C-terminal residues. Similarly, BLAST searches using Dr-CXCL17-like retrieved homologs from certain fish species (Figure 1F and Supplementary Table S3), which feature an N-terminal signal peptide and a mature peptide with six cysteine residues arranged in the CXC-C-CXC-C pattern. All of these fish CXCL17 and CXCL17-like homologs remain uncharacterized and their functions are unknown.

### Preparation of the zebrafish CXCL17s via bacterial overexpression

**Figure 2:**
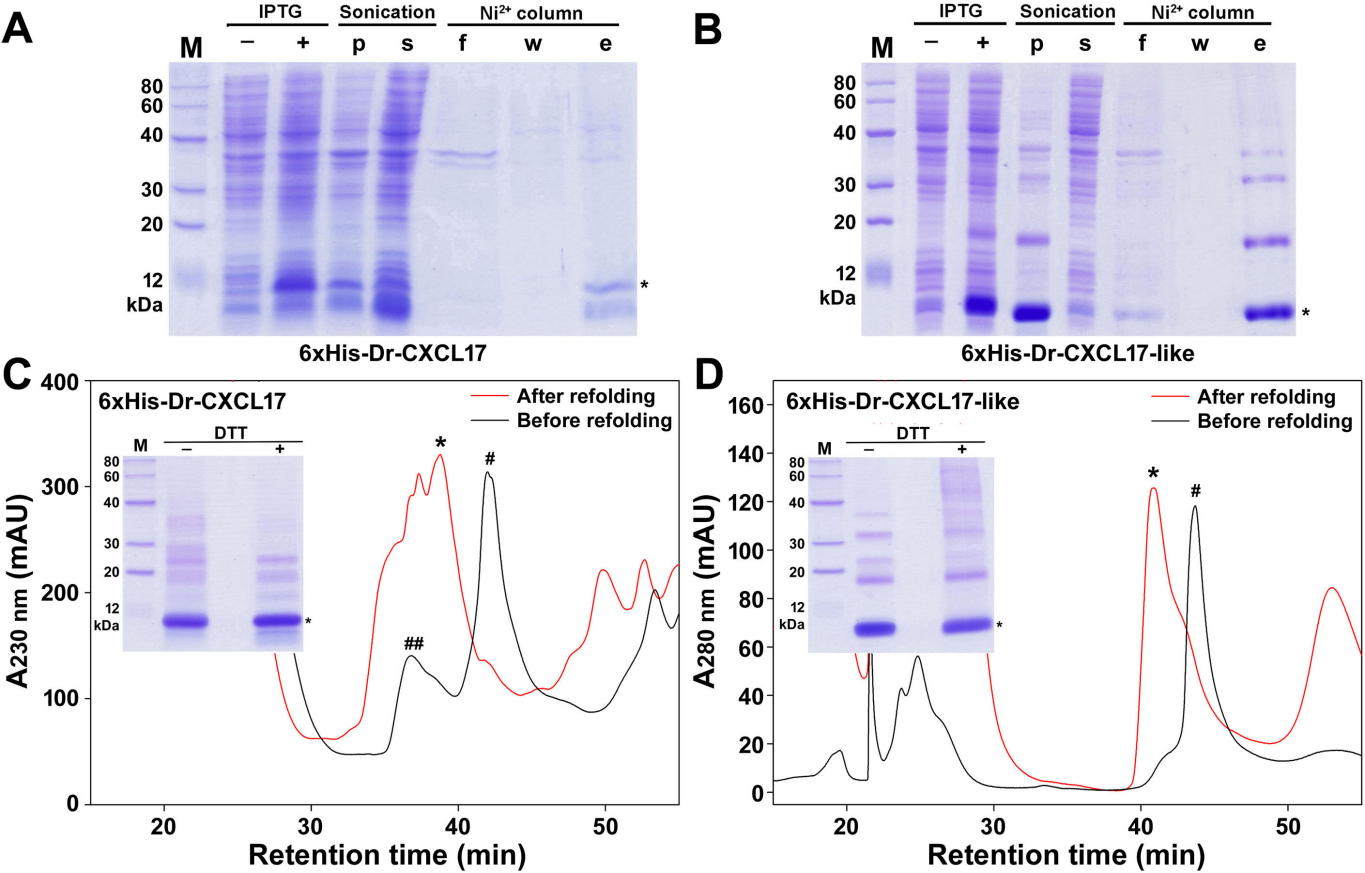
Preparation of the zebrafish CXCL17 and CXCL17-like via bacterial overexpression. (**A,B**) SDS-PAGE analysis of the samples of 6 × His-Dr-CXCL17 (**A**) and 6 × His-Dr-CXCL17-like (**B**). Lane (**M**), protein ladder; lane (‒), before IPTG induction; lane (+), after IPTG induction; lane (**p**), pellet after sonification; lane (**s**), supernatant after sonification; lane (**f**), flowthrough from the Ni^2+^ column; lane (**W**), washing fraction by 30 mM imidazole; lane (**e**), eluted fraction by 250 mM imidazole. After electrophoresis, the SDS-gel was stained by Coomassie brilliant blue R250. Band of the monomeric zebrafish CXCL17 ortholog was indicated by an asterisk. (**C,D**) HPLC analysis of the samples of 6 × His-Dr-CXCL17 (**C**) and 6 × His-Dr-CXCL17-like (**D**). The major peak (indicated by an asterisk) after refolding was analyzed by SDS-PAGE and used for activity assays. **Inner panel**, SDS-PAGE analysis of the major refolding peak. Lane (**M**), protein ladder; lane (‒), without DTT treatment; lane (+), with DTT treatment. After electrophoresis, the SDS-gel was stained by Coomassie brilliant blue R250.

To rapidly produce Dr-CXCL17 and Dr-CXCL17-like for functional characterization, we employed a bacterial overexpression strategy previously used for human CXCL17 in our recent studies [28,30]. For purification purposes, a 6 × His tag was fused to the N-terminus of the mature peptide of each zebrafish protein (Supplementary Figure S5). Sodium dodecyl sulfate-polyacrylamide gel electrophoresis (SDS-PAGE) analysis revealed that both 6 × His-Dr-CXCL17 and 6 × His-Dr-CXCL17-like were expressed in *Escherichia coli* predominantly as inclusion bodies, with particularly high accumulation observed for 6 × His-Dr-CXCL17-like (Figure 2A and B). Following solubilization of the inclusion bodies using an *S*-sulfonation approach, the proteins were purified via immobilized metal ion affinity chromatography (Ni²^+^ column). SDS-PAGE analysis of the eluted fractions showed the expected monomeric form (indicated by an asterisk) along with larger oligomers (Figure 2A and B), indicating a tendency of the zebrafish CXCL17s to undergo intermolecular cross-linking, a phenomenon also observed for recombinant Hs-CXCL17 [28].

The eluted fractions from the Ni²^+^ column were further analyzed by high-performance liquid chromatography (HPLC). For both proteins, a broad major peak (indicated by a hash symbol) was eluted from a C_8_ reverse-phase column (Figure 2C and D, black trace). In the case of 6 × His-Dr-CXCL17, a broad minor peak (indicated by a double hash symbol) was also detected (Figure 2C), likely representing partially degraded products, as suggested by SDS-PAGE analysis (Figure 2A). Following *in vitro* refolding, a broad peak was observed on HPLC for both proteins (Figure 2C and D, red trace). The major fraction (indicated by an asterisk) was manually collected, lyophilized, and analyzed by SDS-PAGE (Figure 2C and D, inner panel). This analysis revealed a prominent monomer band (indicated by an asterisk) along with several faint higher-molecular-weight bands, present with or without dithiothreitol (DTT) treatment, indicating that Dr-CXCL17 and Dr-CXCL17-like are prone to intermolecular cross-linking via isopeptide bonds.

### Activation of the zebrafish GPR25 by the recombinant zebrafish CXCL17s

**Figure 3:**
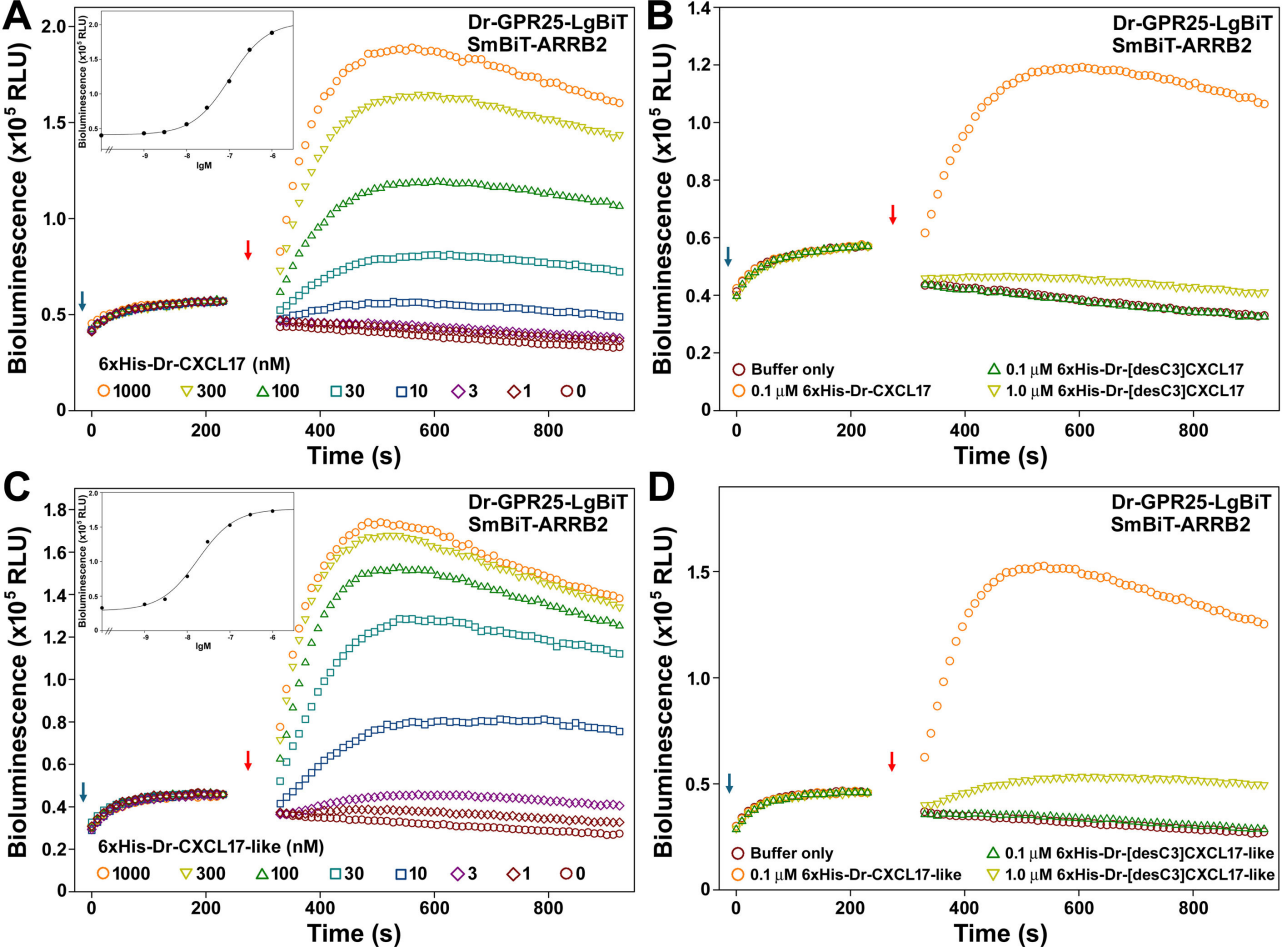
NanoBiT-based β-arrestin recruitment assays of the zebrafish GPR25 induced by recombinant zebrafish CXCL17 or CXCL17-like. (**A,B**) Effect of the WT or truncated zebrafish CXCL17. (**C,D**) Effect of the WT or truncated zebrafish CXCL17-like. In these assays, NanoLuc substrate and different concentrations of WT or truncated zebrafish CXCL17 or CXCL17-like were sequentially added to living HEK293T cells coexpressing Dr-GPR25-LgBiT and SmBiT-ARRB2, and bioluminescence data were measured on a plate reader. Typical bioluminescence change profiles are shown in panels A‒D, and the calculated dose response curves of the WT proteins are shown in inner panels of panel A and C. The blue arrows indicate addition of NanoLuc substrate, and red arrows indicate addition of the zebrafish CXCL17 or CXCL17-like.

To determine whether Dr-CXCL17 and Dr-CXCL17-like function as ligands for Dr-GPR25, we used the NanoLuc Binary Technology (NanoBiT)-based β-arrestin recruitment assay, previously validated with Hs-CXCL17 in our recent study [30]. Upon addition of NanoLuc substrate to living HEK293T cells coexpressing the C-terminally large NanoLuc fragment (LgBiT)-fused Dr-GPR25 (Dr-GPR25-LgBiT) and the N-terminally SmBiT tag-fused human β-arrestin 2 (SmBiT-ARRB2), only low baseline bioluminescence was detected (Figure 3A). Subsequent addition of recombinant 6 × His-Dr-CXCL17 led to a rapid, dose-dependent increase in bioluminescence (Figure 3A), with significant activation observed at concentrations as low as 10 nM. From the dose–response curve (Figure 3A, inner panel), the EC₅₀ value for 6 × His-Dr-CXCL17 activation of Dr-GPR25 was estimated at approximately 100 nM. In contrast, removal of the three C-terminal residues drastically reduced activity: the truncated 6 × His-Dr-[desC3]CXCL17 elicited only minimal bioluminescence increases, even at 1.0 μM (Figure 3B).

For recombinant 6 × His-Dr-CXCL17-like, addition to the NanoBiT-based β-arrestin recruitment assay also produced a rapid increase in bioluminescence (Figure 3C), with significant activation observed at concentrations as low as 3.0 nM. From the dose-response curve (Figure 3C, inner panel), the EC₅₀ value for 6 × His-Dr-CXCL17-like activation of Dr-GPR25 was estimated to be approximately 20 nM, indicating that 6 × His-Dr-CXCL17-like is more potent than 6 × His-Dr-CXCL17. Removal of the three C-terminal residues resulted in a marked loss of activity: the truncated 6 × His-Dr-[desC3]CXCL17-like induced only minimal bioluminescence increases, even at 1.0 μM (Figure 3D).

In summary, the β-arrestin recruitment assay confirmed that both Dr-CXCL17 and Dr-CXCL17-like are potent agonists of Dr-GPR25, indicating that zebrafish GPR25 has two effective endogenous ligands. The three C-terminal residues are critical for the activation of Dr-GPR25 by both proteins, suggesting that Dr-CXCL17 and Dr-CXCL17-like utilize a similar mechanism to engage and activate the receptor.

### Binding of the recombinant zebrafish CXCL17s with the zebrafish GPR25

**Figure 4:**
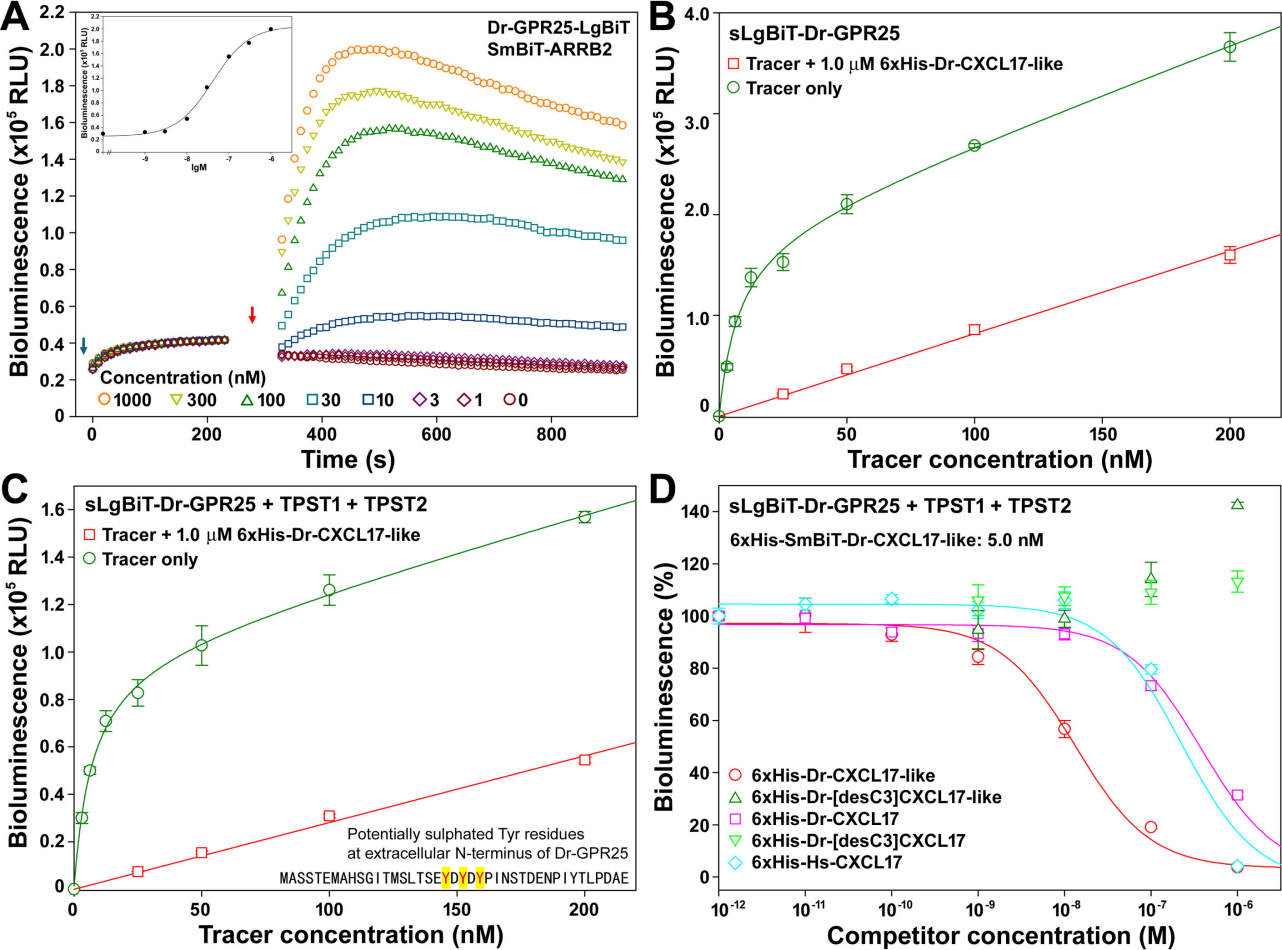
NanoBiT-based binding assays of the zebrafish CXCL17 and CXCL17-like with the zebrafish GPR25. (**A**) Activity of the recombinant 6 × His-SmBiT-Dr-CXCL17-like towards Dr-GPR25 measured via the NanoBiT-based β-arrestin recruitment assay. **Inner panel**, dose-response curve of the tracer. The blue arrow indicates the addition of NanoLuc substrate, and the red arrow indicates the addition of peptide. (**B,C**) Saturation binding of 6 × His-SmBiT-Dr-CXCL17-like with sLgBiT-Dr-GPR25 without (**B**) or with (**C**) coexpression of TPST1 and TPST2. The measured bioluminescence data are expressed as mean ± SD (*n* = 3) and plotted using the SigmaPlot10.0 software. Total binding data (green circles) were fitted with the function of Y = B_max_X/(K_d_ + X) + k_non_X, the non-specific binding data (red squares) were fitted with linear curves. (**D**) Competition binding assays of the recombinant zebrafish or human CXCL17s with Dr-GPR25. The measured bioluminescence data are expressed as mean ± SD (*n* = 3) and fitted with sigmoidal curves using the SigmaPlot10.0 software.

To assess the direct binding of Dr-CXCL17 and Dr-CXCL17-like to Dr-GPR25, we employed a NanoBiT-based homogeneous binding assay, which is highly specific and minimally influenced by endogenously expressed CXCL17 receptors. This proximity-based assay has been validated for several other GPCRs in recent studies [31–34]. To generate an appropriate tracer for the assay, a low-affinity SmBiT tag was genetically fused to the N-terminus of Dr-CXCL17-like (Supplementary Figure S5), as this protein displayed higher activity than Dr-CXCL17 in the β-arrestin recruitment assay. Following bacterial overexpression, purification, and *in vitro* refolding, the resulting 6 × His-SmBiT-Dr-CXCL17-like protein retained high activity in the β-arrestin recruitment assay (Figure 4A), with an EC₅₀ of approximately 40 nM, indicating that the SmBiT-tagged tracer is capable of binding Dr-GPR25.

We next evaluated the tracer in a saturation binding assay (Figure 4B). Incubation of recombinant 6 × His-SmBiT-Dr-CXCL17-like with living HEK293T cells overexpressing the N-terminally secretory LgBiT (sLgBiT)-fused Dr-GPR25 (sLgBiT-Dr-GPR25) produced a hyperbolic increase in bioluminescence. From the resulting binding curve, the dissociation constant (*K*_*d*_) was determined to be 7.5 ± 1.1 nM (*n* = 3). Addition of 1.0 μM unlabeled 6 × His-Dr-CXCL17-like markedly reduced the bioluminescence signal (Figure 4B), confirming that the tracer binds specifically to Dr-GPR25.

The extracellular N-terminus of Dr-GPR25 contains three tyrosine residues that are potentially sulfated, as they are located adjacent to negatively charged residues (Figure 4C, inner panel). When sLgBiT-Dr-GPR25 was coexpressed with human tyrosylprotein sulfotransferases TPST1 and TPST2, enzymes responsible for tyrosine sulfation [35], a hyperbolic saturation binding curve was obtained (Figure 4C), yielding a *K*_*d*_ value of 7.1 ± 0.9 nM. Coexpression of TPST1 and TPST2 did not significantly alter the *K_d_* values, but it slightly reduced the proportion of nonspecific binding (approximately 15% without coexpression vs. around 10% with coexpression at a tracer concentration of 25 nM), suggesting a modest beneficial effect of tyrosylprotein sulfotransferase coexpression on the NanoBiT-based binding assay.

Finally, we performed NanoBiT-based competition binding assays to evaluate the binding potencies of various ligands to Dr-GPR25 (Figure 4D). Recombinant 6 × His-Dr-CXCL17-like efficiently displaced the tracer, resulting in a sigmoidal decrease in bioluminescence, with an IC₅₀ value of approximately 13 nM. In contrast, the recombinant 6 × His-Dr-[desC3]CXCL17-like, lacking the three C-terminal residues, was unable to displace the tracer (Figure 4D), indicating a complete loss of binding to Dr-GPR25. Interestingly, at high concentrations, this mutant caused a slight increase in bioluminescence (Figure 4D). This unexpected effect may result from recruitment of additional tracers to the cell surface by the inactive peptide through interactions with both the tracer and cell surface glycosaminoglycans. Consistent with this explanation, human CXCL17, which is positively charged, has been reported to bind strongly to negatively charged cell surface glycosaminoglycans [36].

Recombinant 6 × His-Dr-CXCL17 was also able to displace the tracer in the competition binding assay, with an IC₅₀ of approximately 380 nM (Figure 4D). This binding potency is about 30-fold lower than that of 6 × His-Dr-CXCL17-like, consistent with its reduced activity in the β-arrestin recruitment assay. Removal of the three C-terminal residues abolished binding, as the truncated 6 × His-Dr-[desC3]CXCL17 showed no detectable interaction with Dr-GPR25 (Figure 4D), in agreement with its poor activity in the β-arrestin assay. Recombinant human CXCL17 (6 × His-Hs-CXCL17) bound to Dr-GPR25 with an IC₅₀ of ~230 nM, comparable with that of 6 × His-Dr-CXCL17, consistent with its previously reported high activity toward Dr-GPR25 in the β-arrestin recruitment assay [30].

### Chemotactic activity of the recombinant zebrafish CXCL17s

**Figure 5:**
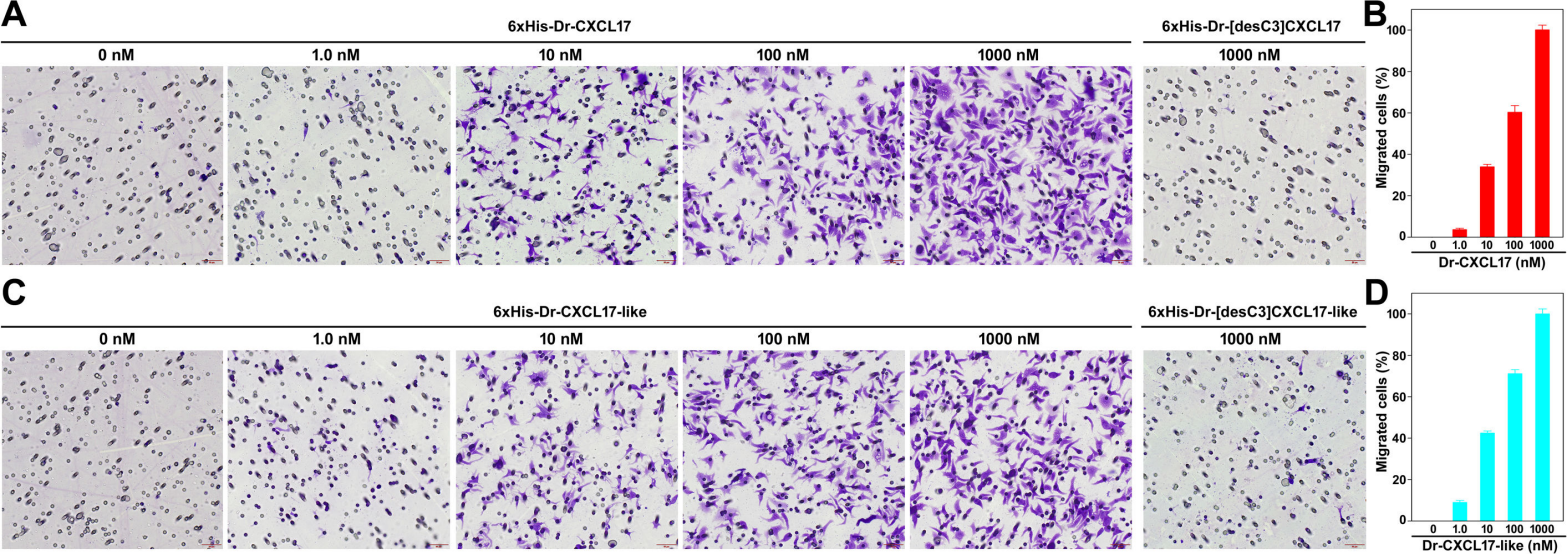
Chemotactic effects of the recombinant zebrafish CXCL17 and CXCL17-like. (**A,B**) Representative images (**A**) and quantitative analysis (**B**) of the Dr-GPR25-expressing HEK293T cells after induced by WT or truncated Dr-CXCL17 in transwell assays. (**C,D**) Representative images (**C**) and quantitative analysis (**D**) of the Dr-GPR25-expressing HEK293T cells after induced by WT or truncated Dr-CXCL17-like in transwell assays. Transfected HEK293T cells were induced to express Dr-GPR25 by Dox, seeded into the permeable membrane-coated inserts, and attracted by chemotactic solution in the lower chamber. After the assay, cells on the upper face of the permeable membrane were wiped off, and cells on the lower face of the permeable membrane were fixed, stained, and observed under a microscope. Representative images of the migrated cells are shown in panel A and C. The scale bar in these images is 50 μm. The migrated cells were quantitatively analyzed using the ImageJ software and the results are expressed as mean ± SD (*n* = 3) and shown in panel B and D.

To evaluate the chemotactic activity of the zebrafish CXCL17s, we performed transwell chemotaxis assays using transiently transfected HEK293T cells expressing Dr-GPR25. As shown in Figure 5, both recombinant 6 × His-Dr-CXCL17 and 6 × His-Dr-CXCL17-like induced dose-dependent chemotactic migration of doxycycline (Dox)-induced HEK293T cells, with significant effects observed at concentrations as low as 10 nM. In contrast, the C-terminally truncated mutants of both proteins exhibited almost no chemotactic activity, even at concentrations up to 1.0 μM (Figure 5), consistent with their reduced activity in receptor activation and binding assays. These findings indicate that both Dr-CXCL17 and Dr-CXCL17-like promote chemotactic migration of transfected HEK293T cells by binding to and activating Dr-GPR25 via their conserved C-terminal fragment.

## Discussion

In this study, we identified two zebrafish CXCL17 paralogs and demonstrated that they are potent agonists of zebrafish GPR25, as confirmed by NanoBiT-based binding and activation assays as well as chemotaxis assays. Owing to their lack of significant amino acid sequence similarity with known mammalian CXCL17s, both paralogs had previously been uncharacterized and their functions unknown. With the rapid advances in DNA sequencing technologies, vast numbers of novel proteins have been discovered across diverse species. The initial step in annotating these proteins is typically to classify them into established protein families based on amino acid sequence similarity with known proteins. However, this approach is ineffective for highly variable proteins, such as the zebrafish CXCL17s reported here, which show no significant overall sequence similarity to functionally characterized counterparts. Consequently, developing strategies to classify and annotate such highly divergent proteins represents an important challenge in the post-genomic era.

Based on three key features derived from mammalian CXCL17s, zebrafish CXCL17s were identified: (1) a C-terminal Xaa-Pro-Yaa motif, where Xaa and Yaa are typically large aliphatic residues; (2) classification as secretory proteins, indicated by an N-terminal signal peptide, with fewer than 200 amino acids; and (3) the presence of six cysteine residues in the mature peptide. These criteria may facilitate the recognition of additional CXCL17 orthologs from other fish species or non-mammalian vertebrates in the future. When combined with amino acid sequence BLAST searches, this approach could enable the identification of more CXCL17 candidates from existing sequence databases. Given that GPR25 orthologs are widely distributed from fishes to mammals, it is reasonable that CXCL17 orthologs are also broadly present among extant vertebrates and remain to be discovered.

CXCL17 has also been reported as an agonist of the MAS-related receptor MRGPRX2 [26]. Our recent findings indicate that human CXCL17 not only activates human MRGPRX2 but also engages the MAS-related receptors MRGPRX1 and MAS1, albeit with slightly lower potency [37]. Notably, activation of these MAS-related receptors by human CXCL17 occurs independently of its conserved C-terminal fragment [37]. In contrast, this conserved C-terminal fragment is essential for CXCL17 orthologs, whether from human or zebrafish, to activate GPR25 orthologs, as demonstrated in the present and previous studies [27,28,30]. These observations suggest that CXCL17 utilizes distinct mechanisms to activate GPR25 and MAS-related receptors. According to NCBI gene database records, GPR25 is broadly distributed from fishes to mammals, whereas MAS-related receptors are restricted to mammals. The identification of CXCL17 orthologs in fishes in this study supports the view that GPR25, rather than MAS-related receptors, represents the evolutionarily conserved receptor for CXCL17. The conserved CXCL17–GPR25 pair likely originated in ancient fishes and subsequently spread across all vertebrate lineages. Our findings provide a foundation for further identification and functional characterization of CXCL17 orthologs in fishes and other non-mammalian vertebrates.

To our knowledge, mammals possess only a single CXCL17 paralog, whereas in this study, we identified two CXCL17 paralogs in zebrafish and several other closely related ray-finned fish species. The zebrafish *cxcl17* gene is located near *cnfn*, *pafah1b3*, and *ceacam1* (Supplementary Figure S2), a genomic arrangement similar to that of the human *CXCL17* gene and its neighboring genes (Supplementary Figure S6). This conservation suggests that CXCL17 orthologs in extant fishes and mammals likely evolved from a common ancestor. In contrast, the zebrafish *cxcl17-like* gene is located on a different chromosome and is flanked by *ponzr3*, *ponzr4*, *ponzr5*, *ponzr6*, *ugt5g1*, and *cldn7a* (Supplementary Figure S3), all fish-specific genes, indicating that CXCL17-like may have an independent origin and be restricted to certain fish species. The evolutionary relationship and physiological roles of fish CXCL17 and CXCL17-like warrant further investigation.

A previous paper mentioned that CXCL17 orthologs from Japanese lamprey and sea lamprey have been documented [38], but in fact, the so-called lamprey CXCL17s are lamprey interleukin-17s (IL17s) [39,40], because the authors mistakenly believed that IL17 was also named CXCL17. The corresponding author of the paper admitted this mistake (personal communication). To the best of our knowledge, CXCL17 orthologs have not yet been identified in non-mammalian vertebrates previously, thus this study represents the first identification and functional characterization of CXCL17 orthologs from non-mammalian vertebrates. Our present study opened the door for functional characterization of CXCL17, CXCL17-like, and their receptor GPR25 in zebrafish and some other fish species. In the future, more studies are needed to disclose their expression patterns in different cell types and tissues under physiological or pathogen-induced conditions, and their *in vivo* functions via gene depletion or overexpression studies.

### Materials and methods

#### cDNA cloning of zebrafish CXCL17s and GPR25

The total RNA extracted from one-month-old zebrafish (Tübingen Strain) was kindly provided by Prof. Cao’s laboratory (Tongji University). The total zebrafish RNA was subjected to reverse transcription using an *EasyScript* ^®^ One-Step gDNA Removal and cDNA Synthesis Kit (TransGen Biotech). Thereafter, PCR amplification was conducted using the reverse transcription mixture as template and synthetic oligoes as primers (Supplementary Figure S4). The PCR products were subjected to agarose gel electrophoresis, and the expected DNA fragments (~400 bp for Dr-CXCL17 and Dr-CXCL17-like; ~1.2 kb for Dr-GPR25) were extracted from the agarose gel and ligated into a cloning vector using a *pEASY* ^®^-Blunt Simple Cloning Kit (TransGen Biotech). After being transformed into Trans1-T1 phage-resistant competent *E. coli* cells, two colonies were subjected to plasmid extraction and DNA sequencing.

### Preparation of the recombinant zebrafish CXCL17s

The DNA fragments encoding the mature peptide of Dr-CXCL17 or Dr-CXCL17-like from the Tübingen strain zebrafish were chemically synthesized using the *E. coli*-biased codons and ligated into a pET vector via Gibson assembly. The resultant expression constructs pET/6 × His-Dr-CXCL17 and pET/6 × His-Dr-CXCL17-like encode the N-terminally 6 × His-tagged zebrafish CXCL17 or CXCL17-like, respectively (Supplementary Figure S5). The expression constructs for the C-terminally truncated mutants were generated via the QuikChange approach using pET/6 × His-Dr-CXCL17 or pET/6 × His-Dr-CXCL17-like as the mutagenesis template. The coding region of the N-terminally 6 × His-SmBiT-fused Dr-CXCL17-like was PCR amplified using pET/6 × His-Dr-CXCL17-like as the template and then ligated into a pET vector via Gibson assembly, resulting in the expression construct pET/6 × His-SmBiT-Dr-CXCL17-like (Supplementary Figure S5). The coding regions of Dr-CXCL17 or Dr-CXCL17-like in these expression constructs were confirmed by DNA sequencing.

Overexpression, purification, and refolding of the zebrafish CXCL17s were conducted according to our recent procedure developed for Hs-CXCL17 [28]. Briefly, the transformed bacteria were induced by isopropyl-β-D-thiogalactopyranoside (IPTG) and then collected via centrifugation and lysed by sonication. The overexpressed 6 × His-Dr-CXCL17 and 6 × His-Dr-CXCL17-like proteins were solubilized from inclusion bodies via an *S*-sulfonation approach, purified by an immobilized metal ion affinity chromatography, and subjected to *in vitro* refolding. The refolded proteins were further purified by HPLC using a C_8_ reverse-phase column (Zorbax 300 SB-C8, 9.4 × 250 mm, Agilent Technologies, Santa Clara, CA, U.S.A.). The eluted fractions from the reverse-phase column were manually collected, lyophilized, and dissolved in 1.0 mM aqueous hydrochloride (pH 3.0) for quantification and subsequent activity assays. The wildtype (WT) or truncated 6 × His-Dr-CXCL17-like and 6 × His-SmBiT-Dr-CXCL17-like were quantified by ultra-violet absorbance at 280 nm using the extinction coefficients (ε_280 nm_) of 7450 M^-1^ cm^-1^ and 8940 M^-1^ cm^-1^, respectively. The WT or truncated 6 × His-Dr-CXCL17 were quantified by SDS-PAGE using 6 × His-Dr-CXCL17-like as standard because they have no ultra-violet absorbance at 280 nm. The samples at different preparation steps were also analyzed by SDS-PAGE.

### Generation of expression constructs for the zebrafish GPR25

The expression constructs for Dr-GPR25 were generated in our recent study [30]. The construct pTRE3G-BI/Dr-GPR25-LgBiT:SmBiT-ARRB2 coexpresses LgBiT-Dr-GPR25 and SmBiT-ARRB2 controlled by a Dox-response bidirectional promoter. The constructs PB-TRE/Dr-GPR25 and PB-TRE/sLgBiT-Dr-GPR25 express an untagged Dr-GPR25 or an N-terminally sLgBiT-fused Dr-GPR25 under control of a Dox-response promoter, respectively.

### The NanoBiT-based β-arrestin recruitment assays

The NanoBiT-based β-arrestin recruitment assays were conducted according to our previous procedure [28,30]. Briefly, HEK293T cells were cotransfected with the expression construct pTRE3G-BI/Dr-GPR25-LgBiT:SmBiT-ARRB2 and the expression control vector pCMV-Tet3G (Clontech, Mountain View, CA, U.S.A.) using the transfection reagent Lipo8000 (Beyotime Technology, Shanghai, China). The next day, the transfected cells were trypsinized, seeded into white opaque 96-well plates, and cultured in complete Dulbecco’s modification of Eagle’s medium (DMEM) containing 1.0 ng/ml of Dox for ~24 h to ~90% confluence. To conduct the β-arrestin recruitment assays, the induction medium was removed, and pre-warmed activation solution (serum-free DMEM plus 1% bovine serum albumin) containing NanoLuc substrate was added (40 μl/well, containing 0.5 μl of NanoLuc substrate stock from Promega, Madison, WI, U.S.A.). Thereafter, bioluminescence data were immediately collected for ~4 min on a SpectraMax iD3 plate reader (Molecular Devices, Sunnyvale, CA, U.S.A.). Subsequently, the recombinant WT or truncated zebrafish CXCL17 or CXCL17-like protein (diluted in the activation solution) was added (10 μl/well), and bioluminescence data were continuously collected for ~10 min. The measured absolute bioluminescence signals were corrected for interwell variability by forcing all curves after addition of NanoLuc substrate to the same level and plotted using the SigmaPlot 10.0 software (SYSTAT software, Chicago, IL, U.S.A.). To obtain the dose-response curve, the measured bioluminescence data at the highest point were plotted with the agonist concentrations using the SigmaPlot 10.0 software (SYSTAT software).

### The NanoBiT-based ligand‒receptor binding assays

The NanoBiT-based homogenous binding assays were developed using 6 × His-SmBiT-Dr-CXCL17-like as a tracer according to our previous procedures for some other GPCRs [31–33]. Briefly, HEK293T cells were transfected with the expression construct PB-TRE/sLgBiT-Dr-GPR25 with or without cotransfection with the tyrosylprotein sulfotransferase expression construct pTRE3G-BI/TPST1:TPST2. The next day, the transfected cells were trypsinized, seeded into white opaque 96-well plates, and cultured in complete DMEM containing 20 ng/ml of Dox for ~24 h to ~90% confluence. To conduct the binding assays, the induction medium was removed, and pre-warmed binding solution (serum-free DMEM plus 0.1% bovine serum albumin and 0.01% Tween-20) was added (50 μl/well). For saturation binding assays, the binding solution contains varied concentrations of 6 × His-SmBiT-Dr-CXCL17-like. For competition binding assays, the binding solution contains a constant concentration of 6 × His-SmBiT-Dr-CXCL17-like and varied concentrations of competitors. To measure cell surface expression level of sLgBiT-Dr-GPR25, binding solution contains 80 nM of synthetic HiBiT peptide. After incubation at room temperature for ~1 h, diluted NanoLuc substrate (30-fold dilution in the binding solution) was added (10 μl/well), and bioluminescence was immediately measured on a SpectraMax iD3 plate reader (Molecular Devices). The measured bioluminescence data were expressed as mean ± standard deviation (SD, *n* = 3) and plotted using the SigmaPlot 10.0 software (SYSTAT software).

### Chemotaxis assays

The chemotaxis assays were conducted using a transwell apparatus according to our previous procedure [28,30]. Briefly, HEK293T cells were transiently transfected with the expression construct PB-TRE/Dr-GPR25 using the transfection reagent Lipo8000 (Beyotime Technology). The next day, the transfected cells were changed to the complete DMEM containing 1.0 ng/ml of Dox and continuously cultured for ~24 h. Thereafter, the cells were trypsinized, suspended in serum-free DMEM at the density of ~5 × 10^5^ cells/ml, and seeded into polyethylene terephthalate membrane (8 μm pore size)-coated permeable transwell inserts that were pretreated with serum-free DMEM (200 μl/well). The inserts were then put into a 24-well plate containing chemotactic agent (WT or truncated Dr-CXCL17 or Dr-CXCL17-like diluted in serum-free DMEM plus 0.2% bovine serum albumin, 500 μl/well). After being cultured at 37°C for ~5 h, the solution in the inserts was removed and cells on the upper face of the permeable membrane were wiped off using cotton swabs, and cells on the lower face of the permeable membrane were fixed with 4% paraformaldehyde solution, stained with crystal violet staining solution (Beyotime Technology), and observed under an Olympus APX100 microscope (Tokyo, Japan). The migrated cells were quantitatively analyzed using the ImageJ software, and the results are expressed as mean ± SD (*n* = 3).

## Supplementary material

Online supplementary material 1

## Data Availability

The data of this study are available in this manuscript, as well as the associated supplementary information.

## Abbreviations

ARRB2: human β-arrestin 2
CXCL17: C-X-C motif chemokine ligand 17
DMEM: Dulbecco's modification of Eagle's medium
DTT: dithiothreitol
Dox: doxycycline
GPCR: G protein-coupled receptor
GPR25: G protein-coupled receptor 25
HEK: human embryonic kidney
HPLC: high-performance liquid chromatography
HiBiT: high-affinity complementation tag for NanoBiT
IPTG: isopropyl-β-D-thiogalactopyranoside
LgBiT: large NanoLuc fragment for NanoBiT
NCBI: the National Center for Biotechnology Information
NanoBiT: NanoLuc Binary Technology
NanoLuc: nanoluciferase
PCR: polymerase chain reaction
SD: standard deviation
SDS-PAGE: sodium dodecyl sulfate-polyacrylamide gel electrophoresis
SmBiT: low-affinity complementation tag for NanoBiT
WT: wildtype
cDNA: complementary DNA
sLgBiT: secretory LgBiT

## References

[1] Pisabarro M.T. Leung B. Kwong M. Corpuz R. Frantz G.D. Chiang N. et al 2006 Cutting edge: novel human dendritic cell- and monocyte-attracting chemokine-like protein identified by fold recognition methods J. Immunol. 176 2069 2073 10.4049/jimmunol.176.4.2069 16455961

[2] Choreño-Parra J.A. Thirunavukkarasu S. Zúñiga J. Khader S.A 2020 The protective and pathogenic roles of CXCL17 in human health and disease: Potential in respiratory medicine Cytokine Growth Factor Rev. 53 53 62 10.1016/j.cytogfr.2020.04.004 32345516 PMC7177079

[3] Xiao S. Xie W. Zhou L 2021 Mucosal chemokine CXCL17: What is known and not known Scand. J. Immunol. 93 e12965 10.1111/sji.12965 32869346

[4] Denisov S.S 2021 CXCL17: the black sheep in the chemokine flock Front. Immunol. 12 712897 10.3389/fimmu.2021.712897 34335630 PMC8320810

[5] Giblin S.P. Pease J.E 2023 What defines a chemokine? - The curious case of CXCL17 Cytokine 168 10.1016/j.cyto.2023.156224 37210967

[6] Lee W.-Y. Wang C.-J. Lin T.-Y. Hsiao C.-L. Luo C.-W 2013 CXCL17, an orphan chemokine, acts as a novel angiogenic and anti-inflammatory factor Am. J. Physiol. Endocrinol. Metab. 304 E32 40 10.1152/ajpendo.00083.2012 23115081

[7] Burkhardt A.M. Tai K.P. Flores-Guiterrez J.P. Vilches-Cisneros N. Kamdar K. Barbosa-Quintana O. et al 2012 CXCL17 is a mucosal chemokine elevated in idiopathic pulmonary fibrosis that exhibits broad antimicrobial activity J. Immunol. 188 6399 6406 10.4049/jimmunol.1102903 22611239 PMC3370106

[8] Burkhardt A.M. Maravillas-Montero J.L. Carnevale C.D. Vilches-Cisneros N. Flores J.P. Hevezi P.A. et al 2014 CXCL17 is a major chemotactic factor for lung macrophages J. Immunol. 193 1468 1474 10.4049/jimmunol.1400551 24973458 PMC4142799

[9] Oka T. Sugaya M. Takahashi N. Takahashi T. Shibata S. Miyagaki T. et al 2017 CXCL17 attenuates imiquimod-induced psoriasis-like skin inflammation by recruiting myeloid-derived suppressor cells and regulatory T cells J. Immunol. 198 3897 3908 10.4049/jimmunol.1601607 28389593

[10] Srivastava R. Hernández-Ruiz M. Khan A.A. Fouladi M.A. Kim G.J. Ly V.T. et al 2018 CXCL17 chemokine-dependent mobilization of CXCR8^+^CD8^+^ effector memory and tissue-resident memory T cells in the vaginal mucosa is associated with protection against genital herpes J. Immunol. 200 2915 2926 10.4049/jimmunol.1701474 29549178 PMC5893430

[11] Hernández-Ruiz M. Othy S. Herrera C. Nguyen H.-T. Arrevillaga-Boni G. Catalan-Dibene J. et al 2019 Cxcl17^-/-^ mice develop exacerbated disease in a T cell-dependent autoimmune model J. Leukoc. Biol. 105 1027 1039 10.1002/JLB.3A0918-345RR 30860634 PMC8279723

[12] Lowry E. Chellappa R.C. Penaranda B. Sawant K.V. Wakamiya M. Garofalo R.P. et al 2024 CXCL17 is a proinflammatory chemokine and promotes neutrophil trafficking J. Leukoc. Biol. 115 1177 1182 10.1093/jleuko/qiae028 38298146 PMC11135614

[13] Yin Y. Mu C. Wang J. Wang Y. Hu W. Zhu W. et al 2023 CXCL17 attenuates diesel exhaust emissions exposure-induced lung damage by regulating macrophage function Toxics 11 646 10.3390/toxics11080646 37624152 PMC10459829

[14] Silver S.V. Tucker K.J. Vickman R.E. Lanman N.A. Semmes O.J. Alvarez N.S. et al 2024 Characterization of prostate macrophage heterogeneity, foam cell markers, and CXCL17 upregulation in a mouse model of steroid hormone imbalance Sci. Rep. 14 21029 10.1038/s41598-024-71137-4 39251671 PMC11383972

[15] Weinstein E.J. Head R. Griggs D.W. Sun D. Evans R.J. Swearingen M.L. et al 2006 VCC-1, a novel chemokine, promotes tumor growth Biochem. Biophys. Res. Commun. 350 74 81 10.1016/j.bbrc.2006.08.194 16989774

[16] Mu X. Chen Y. Wang S. Huang X. Pan H. Li M 2009 Overexpression of VCC-1 gene in human hepatocellular carcinoma cells promotes cell proliferation and invasion Acta Biochim. Biophys. Sin. (Shanghai) 41 631 637 10.1093/abbs/gmp051 19657564

[17] Hiraoka N. Yamazaki-Itoh R. Ino Y. Mizuguchi Y. Yamada T. Hirohashi S. et al 2011 CXCL17 and ICAM2 are associated with a potential anti-tumor immune response in early intraepithelial stages of human pancreatic carcinogenesis Gastroenterology 140 310 321 10.1053/j.gastro.2010.10.009 20955708

[18] Matsui A. Yokoo H. Negishi Y. Endo-Takahashi Y. Chun N.A.L. Kadouchi I. et al 2012 CXCL17 expression by tumor cells recruits CD11b+Gr1 high F4/80- cells and promotes tumor progression PLoS ONE 7 e44080 10.1371/journal.pone.0044080 22952881 PMC3430639

[19] Li L. Yan J. Xu J. Liu C.-Q. Zhen Z.-J. Chen H.-W. et al 2014 CXCL17 expression predicts poor prognosis and correlates with adverse immune infiltration in hepatocellular carcinoma PLoS ONE 9 e110064 10.1371/journal.pone.0110064 25303284 PMC4193880

[20] Koni E. Congur I. Tokcaer Keskin Z 2024 Overexpression of CXCL17 increases migration and invasion of A549 lung adenocarcinoma cells Front. Pharmacol. 15 1306273 10.3389/fphar.2024.1306273 38384293 PMC10879421

[21] Su B.-H. Wang C.-T. Chang J.-M. Chen H.-Y. Huang T.-H. Yen Y.-T. et al 2025 OCT4 promotes lung cancer progression through upregulation of VEGF-correlated chemokine-1 Int. J. Med. Sci. 22 680 695 10.7150/ijms.102505 39898238 PMC11783078

[22] Maravillas-Montero J.L. Burkhardt A.M. Hevezi P.A. Carnevale C.D. Smit M.J. Zlotnik A 2015 Cutting edge: GPR35/CXCR8 is the receptor of the mucosal chemokine CXCL17 J. Immunol. 194 29 33 10.4049/jimmunol.1401704 25411203 PMC4355404

[23] Park S.J. Lee S.J. Nam S.Y. Im D.S 2018 GPR35 mediates lodoxamide-induced migration inhibitory response but not CXCL17-induced migration stimulatory response in THP-1 cells; is GPR35 a receptor for CXCL17? Br. J. Pharmacol. 175 154 161 10.1111/bph.14082 29068046 PMC5740256

[24] Binti Mohd Amir N.A.S. Mackenzie A.E. Jenkins L. Boustani K. Hillier M.C. Tsuchiya T. et al 2018 Evidence for the existence of a CXCL17 receptor distinct from GPR35 J. Immunol. 201 714 724 10.4049/jimmunol.1700884 29875152 PMC6036231

[25] White C.W. Platt S. Kilpatrick L.E. Dale N. Abhayawardana R.S. Dekkers S. et al 2024 CXCL17 is an allosteric inhibitor of CXCR4 through a mechanism of action involving glycosaminoglycans Sci. Signal. 17 eabl3758 10.1126/scisignal.abl3758 38502733 PMC7615768

[26] Ding J. Hillig C. White C.W. Fernandopulle N.A. Anderton H. Kern J.S. et al 2024 CXCL17 induces activation of human mast cells via MRGPRX2 Allergy 79 1609 1612 10.1111/all.16036 38279626

[27] Ocón B. Xiang M. Bi Y. Tan S. Brulois K. Ayesha A. et al 2024 A lymphocyte chemoaffinity axis for lung, non-intestinal mucosae and CNS Nature 635 736 745 736‒745 10.1038/s41586-024-08043-2 39293486 PMC11887596

[28] Hu W.-F. Yu J. Wang J.-J. Sun R.-J. Zheng Y.-S. Zhang T. et al 2025 a Identification of orphan GPR25 as a receptor for the chemokine CXCL17 FEBS J. 292 5998 6015 10.1111/febs.70117 40279398

[29] Zhang J. Wan Y. Fang C. Chen J. Ouyang W. Li J. et al 2018 The orphan G protein-coupled receptor 25 (GPR25) is activated by Apelin and Apela in non-mammalian vertebrates Biochem. Biophys. Res. Commun. 501 408 414 10.1016/j.bbrc.2018.04.229 29727602

[30] Hu W.-F. Wang J.-J. Yu J. Yao J.-J. Liu Y.-L. Xu Z.-G. et al 2025 Human CXCL17 binds and activates fish GPR25 orthologs Biochimie 239 244 251 10.1016/j.biochi.2025.09.009 40972788

[31] Hu M.-J. Shao X.-X. Li H.-Z. Nie W.-H. Wang J.-H. Liu Y.-L. et al 2018 Development of a novel ligand binding assay for relaxin family peptide receptor 3 and 4 using NanoLuc complementation Amino Acids 50 1111 1119 10.1007/s00726-018-2588-5 29770870

[32] Wang J.-H. Li H.-Z. Shao X.-X. Nie W.-H. Liu Y.-L. Xu Z.-G. et al 2019 Identifying the binding mechanism of LEAP2 to receptor GHSR1a FEBS J. 286 1332 1345 10.1111/febs.14763 30666806

[33] Li H.-Z. Wang Y.-F. Shao X.-X. Liu Y.-L. Xu Z.-G. Wang S.-L. et al 2023 FAM237A, rather than peptide PEN and proCCK56-63, binds to and activates the orphan receptor GPR83 FEBS J. 290 3461 3479 10.1111/febs.16765 36853120

[34] Wu H. Hoare B.L. Handley T.N.G. Akhter Hossain M. Bathgate R.A.D 2024 Development of a synthetic relaxin-3/INSL5 chimeric peptide ligand for NanoBiT complementation binding assays Biochem. Pharmacol. 224 116238 10.1016/j.bcp.2024.116238 38677442

[35] Stewart V. Ronald P.C 2022 Sulfotyrosine residues: Interaction specificity determinants for extracellular protein-protein interactions J. Biol. Chem. 298 102232 10.1016/j.jbc.2022.102232 35798140 PMC9372746

[36] Giblin S.P. Ranawana S. Hassibi S. Birchenough H.L. Mincham K.T. Snelgrove R.J. et al 2023 CXCL17 binds efficaciously to glycosaminoglycans with the potential to modulate chemokine signaling Front. Immunol. 14 1254697 10.3389/fimmu.2023.1254697 37942327 PMC10628517

[37] Hu W.F. Wang J.J. Yu J. Liu Y.L. Xu Z.G. Guo Z.Y 2026 CXCL17 activates three MAS-related G protein-coupled receptors independently of its conserved C-terminal fragment Arch. Biochem. Biophys. 775 110666 10.1016/j.abb.2025.110666 41167449

[38] Zhu X. Zhang Z. Ren J. Jia L. Ding S. Pu J. et al 2020 Molecular characterization and chemotactic function of CXCL8 in Northeast Chinese Lamprey (*Lethenteron morii*) Front. Immunol. 11 1738 10.3389/fimmu.2020.01738 33013827 PMC7461807

[39] Tsutsui S. Nakamura O. Watanabe T 2007 Lamprey (Lethenteron japonicum) IL-17 upregulated by LPS-stimulation in the skin cells Immunogenetics 59 873 882 10.1007/s00251-007-0254-2 17924104

[40] Han Q. Das S. Hirano M. Holland S.J. McCurley N. Guo P. et al 2015 Characterization of Lamprey IL-17 Family Members and Their Receptors J. Immunol. 195 5440 5451 10.4049/jimmunol.1500892 26491201 PMC4655163

