# Supplementary material for: Identification and functional characterization of two CXCL17 paralogs from zebrafish
Source: Biochem J. 2026 Jan 22;483(2):135–47. doi: 10.1042/BCJ20253418 (PMC12905490; doi:10.1042/BCJ20253418)
Supplement: Online supplementary material 1 [file bcj-483-2-BCJ20253418-s001.pdf]

Supplementary information

Identification and functional characterization of two CXCL17 paralogs from zebrafish

Jie Yu, Wen-Feng Hu, Juan-Juan Wang, Ya-Li Liu, Zeng-Guang Xu, Zhan-Yun Guo

Research Center for Translational Medicine at East Hospital, School of Life Sciences and Technology, Tongji University, Shanghai, China

Correspondence: Zhan-Yun Guo (zhan-yun.guo@tongji.edu.cn)

Contents:

Table S1: Information about mammalian CXCL17 orthologs aligned in Figure S1.
Table S2: Information about fish CXCL17 homologs identified via sequence blast with Dr-CXCL17.
Table S3: Information about fish CXCL17-like homologs identified via sequence blast with Dr-CXCL17-like.
Table S4: Summary of the possible interactions of the zebrafish CXCL17 and CXCL17-like with the zebrafish GPR25 according to the AlphaFold3-predicted structures.
Figure S1: Amino acid sequence alignment of mammalian CXCL17 orthologs.
Figure S2: Information about the zebrafish *cxcl17* gene (*zgc:158701*).
Figure S3: Information about the zebrafish *cxcl17-like* gene (*si:dkey-112a7.5*).
Figure S4: cDNA cloning of the zebrafish CXCL17, CXCL17-like, and GPR25.
Figure S5: The nucleotide and amino acid sequence of the zebrafish CXCL17 and CXCL17-like overexpressed in *E. coli*.
Figure S6: Position of the human *CXCL17* gene in human genome.

Table S1: Information about mammalian CXCL17 orthologs aligned in Figure S1.

The information was downloaded from the NCBI gene database (https://ncbi.nlm.nih.gov/gene).

Mammalian species	Gene ID	mRNA ID	Protein ID
Homo sapiens	284340	NM_198477	NP_940879
Pan troglodytes	741429	XM_001154726	XP_001154726
Pan paniscus	100975966	XM_003811758	XP_003811806
Macaca mulatta	708108	XM_001105835	XP_001105835
Sapajus apella	116530866	XM_032249326	XP_032105217
Suricata suricatta	115281288	XM_029926676	XP_029782536
Galeopterus variegatus	103586108	XM_008567252	XP_008565474
Mus musculus	232983	NM_153576	NP_705804
Rattus norvegicus	308436	NM_001107491	NP_001100961
Mesocricetus auratus	101826068	XM_021223580	XP_021079239
Octodon degus	101573419	XM_023704517	XP_023560285
Arvicola amphibius	119821858	XM_038340966	XP_038196894
Arvicanthis niloticus	117720566	XM_034519100	XP_034374991
Mastomys coucha	116100415	XM_031384229	XP_031240089
Peromyscus leucopus	114685351	XM_028859975	XP_028715808
Grammomys surdaster	114632694	XM_028781282	XP_028637115
Marmota marmota	107151419	XM_015496800	XP_015352286
Fukomys damarensis	104861740	XM_010623332	XP_010621634
Chinchilla lanigera	102020166	XM_005412354	XP_005412411
Microtus ochrogaster	101982135	XM_005361160	XP_005361217
Heterocephalus glaber	101708913	XM_004873008	XP_004873065
Cavia porcellus	100734695	XM_013146847	XP_013002301
Felis catus	101086020	XM_003997765	XP_003997814
Canis lupus familiaris	111090090	XM_038656810	XP_038512738
Mustela putorius furo	101683082	XM_004780427	XP_004780484
Leopardus geoffroyi	123578900	XM_045442260	XP_045298216
Prionailurus bengalensis	122493991	XM_043598820	XP_043454755
Panthera leo	122207597	XM_042917645	XP_042773579
Puma yagouaroundi	121018500	XM_040457200	XP_040313134
Vulpes lagopus	121484348	XM_041743498	XP_041599432
Hyaena hyaena	120240684	XM_039245502	XP_039101433
Lontra canadensis	116857606	XM_032841857	XP_032697748
Mustela erminea	116580309	XM_032326546	XP_032182437
Ursus arctos	113243523	XM_026482222	XP_026338007
Vulpes vulpes	112931475	XM_026014200	XP_025869985
Puma concolor	112851386	XM_025914921	XP_025770706
Pteropus vampyrus	105307357	XM_011382839	XP_011381141
Panthera tigris	102961962	XM_007086589	XP_007086651
Pteropus alecto	102889606	XM_006903850	XP_006903912
Lutra lutra	125088637	XM_047709872	XP_047565828
Meles meles	123931218	XM_045988111	XP_045844067
Neogale vison	122912075	XM_044257394	XP_044113329
Talpa occidentalis	119249391	XM_037516694	XP_037372591
Sturnira hondurensis	119000497	XM_037065531	XP_036921426
Loxodonta africana	100668516	XM_003406663	XP_003406711
Bos taurus	788717	NM_001083799	NP_001077268
Camelus ferus	102511827	XM_032485980	XP_032341871
Odocoileus virginianus	110123322	XM_020871140	XP_020726799
Bison bison bison	104997641	XM_010852534	XP_010850836
Bubalus bubalis	102401266	XM_006052397	XP_006052459
Capra hircus	102181231	XM_018062511	XP_017918000
Ochotona princeps	101528914	XM_012930594	XP_012786048
Balaenoptera musculus	118885645	XM_036834491	XP_036690386
Halichoerus grypus	118542831	XM_036103355	XP_035959248
Mirounga leonina	117998055	XM_034986582	XP_034842473
Tursiops truncatus	117309080	XM_033845069	XP_033700960
Phocoena sinus	116744452	XM_032614237	XP_032470128
Phoca vitulina	116622190	XM_032388455	XP_032244346
Monodon monoceros	114903747	XM_029236980	XP_029092813
Eumetopias jubatus	114197075	XM_028087908	XP_027943709
Zalophus californianus	113935480	XM_027617446	XP_027473247
Lagenorhynchus obliquidens	113605953	XM_027079714	XP_026935515
Callorhinus ursinus	112807009	XM_025849582	XP_025705367

Physeter catodon	102983204	XM_024132473	XP_023988241
Trichechus manatus latirostris	101348725	XM_004388765	XP_004388822
Orcinus orca	101278327	XM_004271217	XP_004271265
Equus caballus	100629292	XM_003362327	XP_003362375
Choloepus didactylus	119521473	XM_037819798	XP_037675726
Tupaia chinensis	102492896	XM_014582832	XP_014438318
Erinaceus europaeus	103122352	XM_007532948	XP_007533010
Manis pentadactyla	118918052	XM_036896470	XP_036752365
Myotis myotis	118674385	XM_036346119	XP_036202012
Desmodus rotundus	112320178	XM_024577629	XP_024433397
Pipistrellus kuhlii	118702335	XM_036408200	XP_036264093
Molossus molossus	118639378	XM_036275769	XP_036131662
Rhinolophus ferrumequinum	117035523	XM_033129434	XP_032985325
Phyllostomus discolor	114511635	XM_028530396	XP_028386197
Miniopterus natalensis	107534226	XM_016209250	XP_016064736
Rousettus aegyptiacus	107502574	XM_016129904	XP_015985390
Phascolarctos cinereus	110219727	XM_021003326	XP_020858985
Trichosurus vulpecula	118835868	XM_036743155	XP_036599050
Sarcophilus harrisii	116422895	XM_031963247	XP_031819107
Dromiciops gliroides	122745891	XM_043991340	XP_043847275
Vombatus ursinus	114040160	XM_027858170	XP_027713971
Ornithorhynchus anatinus	103166888	XM_029066640	XP_028922473
Tachyglossus aculeatus	119946135	XM_038767563	XP_038623491

Table S2: Information about fish CXCL17 homologs identified via sequence blast with Dr-CXCL17.

The protein sequence blast was conducted via the NCBI online server (https://blast.ncbi.nlm.nih.gov/Blast.cgi), and information about the retrieved homologs was downloaded from the NCBI gene database or nucleotide database.

Fish species	Gene ID	mRNA ID	Protein ID	Amino acid sequence
Danio rerio	100151367	NM_001144821	NP_001138293	MKTMNFQILVLAFAVMIVTNIQCEARPQEGKSDKSAEVKGHAMPRKCNCQVRGTALDRNCVCEMPHKSRPTLNPEQKNMCLKKKIKTFRKCLQFMGANKKIAKGASLPI
Danio aesculapii	130242767	XM_056474668	XP_056330643	MNFQILVLAFAVVTVTNLQCQARPQVENSDKSAEVKGHATPRKCNCRGRGTALDQNCVCEMPPKSRQTLSPEQKNMCLKKRTRTFRKCQQLIGANKKSAKAISMPI
Ictalurus furcatus	128607944	XM_053625239	XP_053481214	MKFVMLLLVFTAMVGFSALCQAVIDSEVSDKNVTASSGDAVGPADVTSHTRSTKQRGANCACAGKSKAQRGCQCKMNNFKHGLSPEERVLCLKKGIRNYKKCKSVILKTVKTKEDPKQISIPV
Ictalurus furcatus	128607944	XM_053625248	XP_053481223	MKFVMLLLVFTAMVGFSALCQVIDSEVSDKNVTASSGDAVGPADVTSHTRSTKQRGANCACAGKSKAQRGCQCKMNNFKHGLSPEERVLCLKKGIRNYKKCKSVILKTVKTKEDPKQISIPV
Ictalurus punctatus	108266547	XM_017470017	XP_017325506	MKFVMLLLVFTAMVGFSALCQAVIDSQVSDKNVTASSGDAVGSADVTSHTRSTKQRSANCVCAGRSKARRGCQCKMNNFKYGLSPEERVLCLNKGIRNYKKCKTVILKTVRTKEDPKQISIPM
Ictalurus punctatus	108266547	XM_017470026	XP_017325515	MKFVMLLLVFTAMVGFSALCQVIDSQVSDKNVTASSGDAVGSADVTSHTRSTKQRSANCVCAGRSKARRGCQCKMNNFKYGLSPEERVLCLNKGIRNYKKCKTVILKTVRTKEDPKQISIPM
Labeo rohita	127178215	XM_051130938	XP_050986895	MNFQILLLAFAVVIATNIHCQAAQPQLRDSDKSPFPKGLAIFKQQGKKCNCLGIRDPLNQNCHCEMQRQLRMKEQKTLCLKNGILSKKCLELAGGNRKERKGFSMPI
Labeo rohita	127178215	XM_051130939	XP_050986896	MNFQILLLAFAVVIATNIHCQAQPQLRDSDKSPFPKGLAIFKQQGKKCNCLGIRDPLNQNCHCEMQRQLRMKEQKTLCLKNGILSKKCLELAGGNRKERKGFSMPI
Pseudorasbora parva	137083191	XM_067448952	XP_067305053	MKVQVILLAFAVVVATNIHCQVQSQLTDADTSPVFKGHVMSRQVWKCNCNGRRISLEQNCPCELQRQNRMLSKEQRAVCQKKGIVTYKKCQQLTGGNRKEKNVRKVYSMPI
Carassius carassius	132158314	XM_059567675	XP_059423658	MNFQVPLLVFAVVITTSINCQEQPQVGESNKSPVVKGQVISRHQGKTCNCIGKRNALEQNCPCGLQRQNRILSNDKKAWCQKKGISTFKKCQQLIGENRKEKKGISMPI
Onychostoma macrolepis	131522610	XM_058748243	XP_058604226	MNFQLLLLVFAVVIATNIHCQAQPPLGNSNKSPAVKGQVISRQQGKTCNCTGRRNALEQNNCPCELQRQYRILSKEQKALCLKKGISTFKKCQQLIGGNRKEKKGFSMPI
Carassius gibelio	128030755	XM_052618693	XP_052474653	MRNFQVLLLVFAVVIATNIHIQAKPQLGDSDKSPVVKSKQQSKPCSCVGRRNALEQDNCTCERQRQHGTLSEEQRTCQKKKYRKCPRLNRGNKKEEKGISMPI
Myxocyprinus asiaticus	127452759	XM_051718450	XP_051574410	MKLQVLLLAFAVFIASVHCQAQAQLGQSHKSPVIKGQVLLSRQQGRSCNCSGRRNGLELNCPCELHRQHRVLSQKQWALCQKKLIQTYKKCQQMIFGEKRKEKKGNKGISNPI
Megalobrama amblycephala	125243866	XM_048153711	XP_048009668	MKFHMLLLAFVVVIVTNIHCQVQPQLEDSDKSPVDKGHVMSKRQVRTCNCGGRRNALEQNCPCERQRQYEILSKEQRAFCQKKGIWTYKKCQQMIGGNRKEKKGNKGFSMPI
Puntigrus tetrazona	122360472	XM_043261100	XP_043117035	MNFQVLLLVCAVVIATNIHCQAQPQLGDSNKSPVVKGHIISVQQGKKCNCIGRRNALEQNYCPCEQQRQYKLLSKEQTFACLKKGISTFKKCQQFTGGNKKKKVISTPF
Pimephales promelas	120484472	XM_039679646	XP_039535580	MKFQVLLLAFALVIATNIYCQAQPQRRDSDKSPEVSRRQVRTCNCSGRRFENCPCELQRQYRVLKEQKAFCQKKGSATSKICQRLTGGIRKQKKGNIGSMPI
Paramisgurnus dabryanus	141280008	XM_073811699	XP_073667800	MKFQILLLTCAVLIATDVYGEAQPQQGDSDKSMSLQQGRPCNCIERRNGQKQNCPCSLPSQRTILSEKQRDLCKKKGIKTFKKCKQLIPQNIKNGKKSNKAMGTPF
Cirrhinus molitorella		KAL1259315	QQF64_009892	MMNFQVLLLAFAVVIATNIHCQALPQLGDSNKSPVVEGLTISRQPSKLCNCIGRRNALDQNYCTCEMQRQHRMLNKEQIALCLKKGISTYKKCLQWTGGNRKDRKAISMPI
Cirrhinus molitorella		KAK2903191	Q8A67_007904	MNFQVLLLAFAVVIATNIHCQALPQLGDSNKSPVVEGLTISRQPSKLCNCIGRKNALDQNYCTCEMQRQHRMLNKEQIALCLKKGISTYKKCLQLTGGNRKDRKAISMPI
Culter alburnus		KAK9960771	ABG768_008606	MKFQVLLLAFVVVIVTNIHCQVQPQLEDSDKSPVDKGHVMSKRQVRTCNCGGRRNALEQNCPCELQRQYKILSKEQRAFCQKKGIGTYKKCQQMIGGNRKEKKGNKGFSMPI
Anabarilius grahami		ROI36443	DPX16_11384	MKFQVILLAFAVVIVTNIHCQAQHQLEDSDKSPVDKGHIMSKRQVRTCNCRGKRNALEQNCPCELQRQYGILSKEQKAFCQKKGIGTYKKCQQRTGGNRKEKKGNKGFSMPI
Leuciscus waleckii		XDV40076	PO909_009229	MKFQMLLLAFALVIATNIHCQAQPQLRDSDKSPEVKGHVMSRRQVRTCNCSGRRISVEQNCPCAQRLLSKEQRALCQKKGIASFKKCQQLNGGIRKQKKGNISMPI
Cyprinus carpio		KTF95749	cypCar_00039330	MNFQVLLLVFAVVIATNIHCQAQPRLGDSDKSPVDKSKQQGKTCNCTGRQNALEQNNCPCESQYRILSKERAVNERALCQKKGILTKKCQHMNRSNRKEKKGISMPI
Phoxinus phoxinus		KAK7147560	R3I94_010169	MKFQVLLLAFALVIATNIHCQAQPQLRDSDKSPEGNGYIMSRRQVRTCNCNGFLSKEKKAFCQKKGIATSKKCKQLTGGNRKQMKANNGPSMP

Table S3: Information about fish CXCL17-like homologs identified via sequence blast with Dr-CXCL17-like.

The protein sequence blast was conducted via the NCBI online server (https://blast.ncbi.nlm.nih.gov/Blast.cgi), and information about the retrieved homologs was downloaded from the NCBI gene database or nucleotide database.

Fish species	Gene ID	mRNA ID	Protein ID	Amino acid sequence
Danio rerio	100536854	NM_001386806<br>XM_073906074	NP_001373735<br>XP_073762175	MTKPICLVFALLILTIILCNNSVCSQRRSMKQSAVCGCKLYPDKGLKCTKRPNPKSRDE<br>YYEILKCICRDTQIFSKSSRKEYLKRCNKFYPSLPL
Misgurnus anguillicaudatus	129439872	XM_073859897	XP_073715998	MSKTIYLVFVLVILTTLLCNSPVCSQRGSGNGQATCGCKIHPRKGLQCVRRPHTTWAEI<br>VQCICNNRKTYLNGDSKRLYQKYCNRTISTPL
Chaetodon trifascialis	139351666	XM_070993653	XP_070849754	MSRIIVVSLLLIILVDNFYHSTASHAEFRVSKVHIRKCRCRVFPDGRIKCRSPLLPSNY<br>VKKQDLIKCFCNKSNQHKFPEFQAACGSWRPNIPITLL
Garra rufa	141331768	XM_073836795	XP_073692896	MSKPICLLFVLVILTTILCYNPVCSQRGSLKQKTACGCKIHPNGSLWCAKRHNPKNSYE<br>YDEVVRCICRNPTKYLNENSKKQFVRMCHSKYPSLPL
Oncorhynchus clarkii lewisi	139417144	XM_071166389	XP_071022490	MSKLICVALLLVFLVSIWCHNTVSSTKWSVRDVLRKCRCKVLPNGREICRKPLFPKTPE<br>ETKQLIKCFCRKYKLYKHSAKLNLQTKRDLEKCSFIWSNPF
Oncorhynchus mykiss	110531667	XM_021614986	XP_021470661	MSKLICVALLLVFLVSIWCHNTVSSTKWSVRDVLRKCRCKVLPNGREICRKPLFPKTPE<br>ETKQLIKCFCRKYKLYKHSAKLNLETKRDLEKCSFIWSNPF
Oncorhynchus keta	118368844	XM_035753275	XP_035609168	MSKLICVALLLVFLVSIWCHNTVSSTKWSVRDVLRKCRCKVLPNGREICRKPLFPKTPE<br>ETKQLIKCFCRKYNLYKHSAKLNLETKRDLEKCSFIWSNPF
Oncorhynchus kisutch	109906877	XM_031791963	XP_031647823	MSKLICVALLLVFLVSIWCHNTVSSTKWSVRDVLRKCRCKVLPNGREICRKPLFPKTPE<br>ETKQLIKCFCRKYNLYKHGAKLNLETKRDLEKCSFIWSNPF
Leuciscus waleckii		XDV19005	PO909_024587	MSKPICLVFALLILTTILCNNSVCSQRGSWKQSAVCGCKIHHDNSLRCTKKHSPKTLEE<br>YHKMVGCICSDRQKYFNSKKQFLNMCKSYSQTPL
Cirrhinus molitorella		KAK2913578	Q8A67_001977	MSKPICLLFVLVILTTILCYNPVCSQRGSLKQIAACGCNFHPNGSLRCTKRHNLKTGYE<br>YNEVVKCICRNPTKYLNEDLKKTFVRMCNKLSMPL
Phoxinus phoxinus		KAK7171077	R3I94_001092	MSKPICLVFALLILTTILCNNSVCSQRGSSKQSAVCGCTIHHNSLRCTKRHRPKTIEEN<br>HKMISCICNNPQKYLNEASKKQFRNMCISNSQTPF
Phoxinus phoxinus		KAK7177025	R3I93_001084	MSKPICLVFAVLILTTILCNNSVCSQRGSSKQSAVCGCTIHHNSLRCTKRHRPKTIEEN<br>HKMISCICNNPQKYLNEASKKQFRNMCISNSQTPF
Triplophysa rosa		KAI7806138	IRJ41_001095	MSKTVSLVFVLVILTTILCNNSVCCRGFWKGRISCGCKIHPEKGLQCSKRHNFKTMDEI<br>MKCICRNPRTYLTDSSKRLFQKMCKPNISTPL
Megalops atlanticus		KAG7465314	MATL_G00175080	MSKLCCGSLLLILLAVIWCDYSVESRRWISPNRLLKDECKCRVLANKKVSCRKDFSPKT<br>MQERFDMLKCLCKKHYNELEMKMDADFQKACKKIFRDVPIPQPLS

Table S4: Summary of the possible interactions of the zebrafish CXCL17 and CXCL17-like with the zebrafish GPR25 according to the AlphaFold3-predicted structures.

The AlphaFold3 prediction was conducted via the online server (https://alphafoldserver.com). The receptor residues involving ligand-binding are indicated by asterisks in Figure 1B.

Ligand	Ligand residue	Interacting residues in Dr-GPR25
Dr-CXCL17	C-terminal carboxyl moiety	ε-amine moiety of K274 (TMD6)
	I109	I127 (TMD3); F270 (TMD6); F307 (TMD7)
	P108	W104 (TMD2)
	L107	L299 (TMD7); M300 (TMD7)
Dr-CXCL17-like	C-terminal carboxyl moiety	ε-amine moiety of K274 (TMD6)
	L95	I127 (TMD3); F270 (TMD6); F307 (TMD7)
	P94	W104 (TMD2)
	L93	L299 (TMD7); M300 (TMD7)

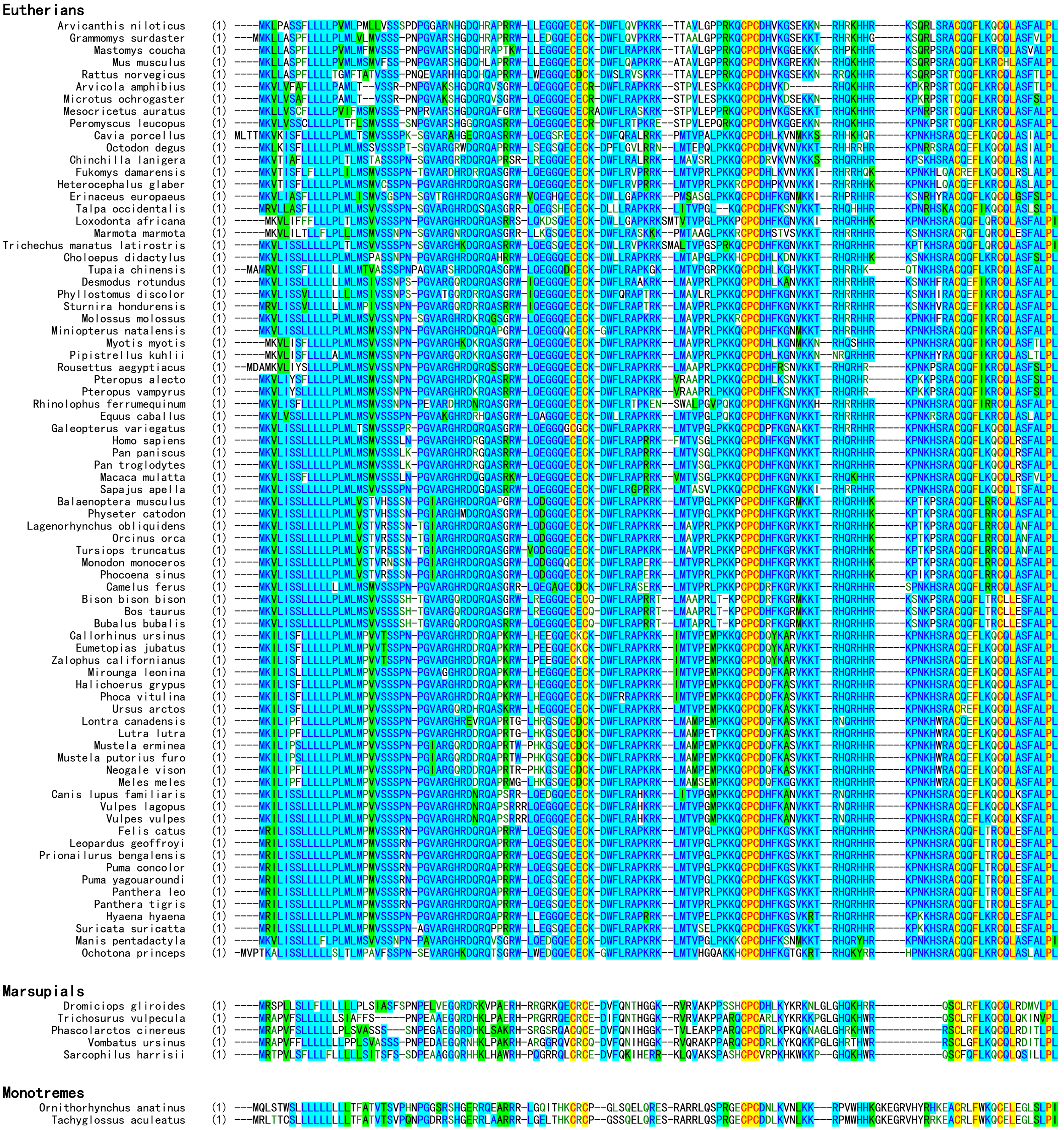

Figure S1: Amino acid sequence alignment of mammalian CXCL17 orthologs.

Accession numbers of these orthologs are listed in Table S1. These sequences were aligned via AlignX algorithm using the Vector NTI 11.5.1 software.

A (Gene position and architecture)

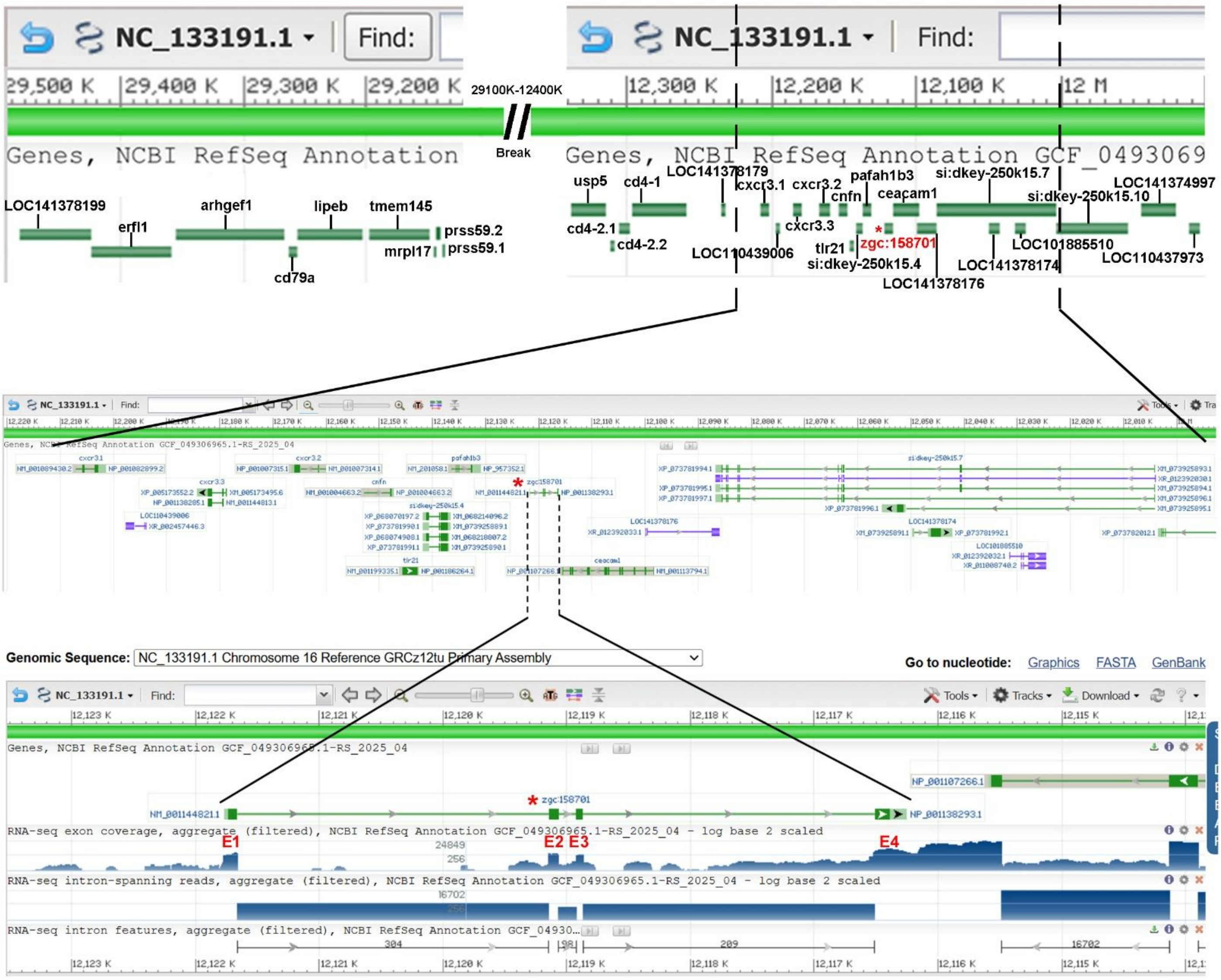

B (genomic DNA sequence, exons in red and highlighted in yellow, coding region underlined)

ataatctctttctccttcccaaattgcacgcagctcttatcacagatcttgacagttctcagcaaatgagaaacactgtcaacatttcttttacacttcatttctaaaagtctatc
cggcaagttgaggcattatcagacgtgacttttgagactcagatatcagagctttgtcatataaatactgaaactgtagatctgaacactgaagacttaaaagtcgactttttagac
agtttgtctcattttgattctgattgccgatgaaaaccatgaacttccagatacttgtgctggcttttgctgtgatgattgtcactaacatccagtgtgaaggtaaacatttttgc
tgaatttatattaatattattattagtgaaaagttaagaaaaatattattattattattattattttactttttgaaagaagttatgcttatgaaggctgtatttatgtgataaa
gtattattgtaaaatattattttaatttaaaataactcaaactttatttaaaattaaaattttagctttaataatcaagtgtttagtggcagataagtattcaaaaaatgctcaat
aaacaaataaaattatttaatcaaataaaatttttaaattctttttagtatgtccaaatttgaaaacttaaaataagattataatatttgctataaatgtgtttagtcaacataaa
aaaaaaaaaaaaactaatttaacattttgaaagaatatttttttgcttcgactgtcttacaaactgaaactaaaataattttgttcattcatactttttttttttttcggcttagtcc
ctttattaatcagggattgccacagtgtaatgaaccgccaacttatccagcgtatgttttatgcagcggatgccctcccaactgcaattcatcactgggaaacacccatacactcc
ccttaacacacatacactacagacaattcagcttacccaattcatatagcgcatgttcttggacttgtgggggaaaccggagccgccccatataatttacatcaattataaagttg
agtcaaattagcgttgccacctcacagcaagaaggtcactggttcgagccttggctgggtcagatggcatttctgtgtggagtttgcatgttctctgtgtttgtgtgggtttcctg
tctttacagttcccacacaactgtaaagatatgcgctatataggtgaattgggtaggctaaatttgtttgtagtgtatttgtgtgaatgcgagtgtatgtgtttttcccagtgacg
ggttgcagctaaaagggcatccgctgtgtaaaacatactgtatgctggattagttggggattcatttacgttgtggcgacacctaattaataaagagacgatgccgaaattttttt
tttaatgaattaataaactgtatatttatattatatttacatagttatatctatataatatatctatataatatatttgctcttccagctatttactctcctctctatatatttgc
tcttccagctaatatttgtgctcttccagcattttactgtgaattattattaaagatcaagcaacagtgaaaacaatatagcagcaaatcaacatgttacaattaattctgaaaaa
acatatctacaaaatttagcattgtgcatggttatgtgttaatataaatatacataaataatctgaaataaagttgaaggacagttaaagtcagaattattagccccccctttgaat
ttgtttttctttttaaaatttttcccaaattatgtttagcggagcaaggaaatgttcacagtatgtctgatgatatttattcttctgcaaaaaatcttatttgttttactttggct
agaataaaagaagttataaatttttttaaacaccattttaaggacaaaattattagcccctttaagctatttttttccgatagtctacagaagaaaccctctttatacaataacttgc
ctaattactctaacctgcctagttaaccaaattaacctagttaagcctttaaatgtcactgtaagctgtataaagtgtcttgaaaaatatctaagatattatttactgtcatcatg
gcaaatatataataaattagttactagaaatgagttattaaaactattatgtttagaaatgtgttgaaaaaatcttctctccgttaaacagaaattgggaaaaaaattaatggggg
gcaaataattcaggggggctgtatgtgaataaaaagtttctttttttcccatcccagtacacctcatttcgaaaatgaataatttatccaatatgcagttaatacaggacatgaatt
ttggtgcatttaaacaaaataaatttattaaacatgtttgttaagatatgtttgttaaaataatattttattcaccaaacatgtttagaaattgaaatataatacaatttaactct
aggaaaatattgcaaaaaaataaaataaataataataataatacaacctataaatttcaacaacatttttcatatttgctttgatttgtcctctttttttaaatttgtatttaatt
ttttctaaaacatagacattttggtgtactattttttgaactgttatcataaggtattttgttagatgagctccagatttggcttcagtactgactaatctaatgtatatgcacaaa
gataatattgtatagcttcctattaaaatacgaatttaaaagagagatttgtgagggacgtactcatatgctcagcactgtatcgtatatgccaaagaaatatttctaattctcat
aatattttgaattcattattaatactattatcattctttttttctaatacaatttctgttttatgcagcacgacctcaagagggaaagtcagacaaatctgcagaggtcaaaggcca
tgctatgcctaggaaatgcaactgccaaggtaagtggtgaagtactttctcctgtcatcaaaaaagaaaacaagaagccctaatgtgctgcttaaagtgttgtgagacattcaagt
gcttataatgtaaaatgagagtcctttctttctgactgatgtgttgttttgtgtagtgaggggaactgctctggatcggaactgtgtctgtgagatgccacataaatctagtaagt
caaacaagatgcttttaccaagatatctgtctgtttggtaagtaaatgtacaacaaacagccttataaaaatctgcataaaaaatcaggcgtgtgaaaatgattttttcgccccatt

```
tggtatgtaattatgaggtttaaatactccatttagtaacattttaagcactatttttagctagattagcttgttggatggtaatcaaaccaaaaatattttctaaagatttagaa
tcatatatatatatatatatatatatatatatatatatatatatatattttttttttttttttaaatacaaatttattacattatatatcattcaggctcccgtttcccccacagtg
caaagacatgcgctatagatgaattgaataagctaaattggccatagtatatgtgtgtgaatgagtgtgtatgggtgtttcccagtgtttggttgcggctggagtggtatgagctg
cgtaaaacataggctggataagtttgtggttcattctgctgtggcgacccctgattgataaagggactaaaccaaaaagaaaatgaatgaataaatatatcattcagaaaaaaaaa
ttgtagtctattgctatttattaggcaaataacaaactggacagcacaatcaactttttttcctcagttagaatttggccatttcaataaaaaaaataaaaaaattaaaacatcatt
ttaatgatgtagaacttcattattttttttctagcctctcttgtaaagaagtaaattggaaaattcatggataacaacaaaagcctaattagtattaaaattaactttaatataggc
cgcttttcgtgctttgcatattttcattgattatttttttctttcaagaataagttccaagatttagaataaaagttctttgtaaaatgttttcggtcacactttacaataaggttc
gttcattagttaatgtttattaatgcattttctaacatgaacaaacaatgaacgatacatttactactttattggttcatgttagttaacgttagttaatgaaaatataatagttc
attgttagttcatgttaactcatggtgcattagctaatgttaacaagcacagacttgaatgttaataatgcattagtaaatgttcaattatgattaattaatgctgtacaagtgtt
gttcatgattagtttatgttaataaatgcattaactaatgaaccttattgtaaagtgttaccatgttttctgtatttaacaatagagcaggtcagtagtgaaagctgattttactt
acatttgattctgcctggtcttcaagtcttcgataggttatttccacaattgattatggcatagtagtcaaaataggacaaatgagctcatgtattgggcaaattctggatctttg
tcagtgggaggtgagtctttcgaaccacccaaacctcccctggctaagggtgtgataaggaaaatgatgcattaaatggtcacttcctttgcttgttatatttactataatgcttt
aaggtcaggtataattaatcgtttttttgattatacagacaaatatgtgatttgtgaatattttgatgattcatgtttttacccagttaaaggcaggttagtctttttttcttttttta
ttgaataaaggtcagtatcactactatactgccaataatatttctattaagtcacattgagctgtaccttttgtaccgtttagtgataaggactgaacaagtggtttttgatttct
acttggccagccaaaaaggttttagacccataataacctgtattttaataaataaggttacccttaccaaaaatttacataaaatccaatccataattctcaattatttcattcat
tcatttcttttcagcttaatcactttattaatccagagttgccacatgcagctgaattaatccgccaacttatccagcacatgtttttacacagcagatgcccttccatctgcta
ttctcatttagtcaattcaattccaaacaaacactgctagcctaactaatataggttttacaatttttatacaagtattattaggaaaagaataaataaactagctgcaaatgata
ctatattaataataaaggtttttttgctctgattttgcaaaaaaaaaattgttctttgtaaaaaaaaagggttatataggttattcaaatcttcacagaaactggttcattttaactg
aagggttttcactgattagctgggagttcttctatggcaacattgcaataaataaatctttgtatcttcatgtttatgtgtattattttgaaacgaacagccagtattgaaataag
atttttatttattaatttacctttattttcaggaccaaccctcaacccagagcagaaaaacatgtgcttaaagaagaagattaagacatttagaaaatgcctgcagtttatgggtg
caaacaagaagatcgccaaaggagccagtttgccaatataagaccttttggcattcactgtcacacagaaagttatacatcttttacacaatacctatagcagtgcatactgaagt
cgtgtatactcaactatttttgtatctgtattattatgtaacatttattcagtccagtttctaacttcagattttaaaaagttatatattggataaagagatttaaatgaattaa
aaataaaacatgctgataaaagagagcagttcatttgaataatgtgcttttatttaattttattaatgaaaatatatgagatttcacatttttttgatcttcggaataatcattttg
aatatacttaaacactttgcattatatttctttgaggaaagatttttttgattccaagatatgagaaacaacagtgaagcagaaaggtagctaggatagttgctctttcaggtaagt
catttgcttaaatcagatgtcagcttttccaaacaaaaaactacaaacggaaataagccagcctctctaaagttcagcttttcacaaatgaataaaattagagtaccaagttaaat
aactgatcacttatagttcttagaaataaatatataaaaaaaataatgcagta
```

C (cDNA sequence and encoded amino acid sequence)

```
1      T AGA CAG TTT GTC TCA TTT TGA TTC TGA TTG CCG ATG AAA ACC ATG AAC TTC CAG ATA CTT GTG CTG GCT TTT GCT GTG ATG ATT GTC
       A TCT GTC AAA CAG AGT AAA ACT AAG ACT AAC GGC TAC TTT TGG TAC TTG AAG GTC TAT GAA CAC GAC CGA AAA CGA CAC TAC TAA CAG
                                                  M   K   T   M   N   F   Q   I   L   V   L   A   F   A   V   M   I   V

89    ACT AAC ATC CAG TGT GAA GCA CGA CCT CAA GAG GGA AAG TCA GAC AAA TCT GCA GAG GTC AAA GGC CAT GCT ATG CCT AGG AAA TGC AAC
      TGA TTG TAG GTC ACA CTT CGT GCT GGA GTT CTC CCT TTC AGT CTG TTT AGA CGT CTC CAG TTT CCG GTA CGA TAC GGA TCC TTT ACG TTG
       T   N   I   Q   C   E   A   R   P   Q   E   G   K   S   D   K   S   A   E   V   K   G   H   A   M   P   R   K   C   N

179   TGC CAA GTG AGG GGA ACT GCT CTG GAT CGG AAC TGT GTC TGT GAG ATG CCA CAT AAA TCT AGA CCA ACC CTC AAC CCA GAG CAG AAA AAC
      ACG GTT CAC TCC CCT TGA CGA GAC CTA GCC TTG ACA CAG ACA CTC TAC GGT GTA TTT AGA TCT GGT TGG GAG TTG GGT CTC GTC TTT TTG
       C   Q   V   R   G   T   A   L   D   R   N   C   V   C   E   M   P   H   K   S   R   P   T   L   N   P   E   Q   K   N

269   ATG TGC TTA AAG AAG AAG ATT AAG ACA TTT AGA AAA TGC CTG CAG TTT ATG GGT GCA AAC AAG AAG ATC GCC AAA GGA GCC AGT TTG CCA
      TAC ACG AAT TTC TTC TTC TAA TTC TGT AAA TCT TTT ACG GAC GTC AAA TAC CCA CGT TTG TTC TTC TAG CGG TTT CCT CGG TCA AAC GGT
       M   C   L   K   K   K   I   K   T   F   R   K   C   L   Q   F   M   G   A   N   K   K   I   A   K   G   A   S   L   P

359   ATA TAA GAC CTT TTG GCA TTC ACT GTC ACA CAG AAA GTT ATA CAT CTT TTA CAC AAT ACC TAT AGC AGT GCA TAC TGA AGT CGT GTA TAC
      TAT ATT CTG GAA AAC CGT AAG TGA CAG TGT GTC TTT CAA TAT GTA GAA AAT GTG TTA TGG ATA TCG TCA CGT ATG ACT TCA GCA CAT ATG
       I   *

449   TCA ACT ATT TTT GTA TCT GTA TTA TTA TGT AAC ATT TAT TCA GTC CA
      AGT TGA TAA AAA CAT AGA CAT AAT AAT ACA TTG TAA ATA AGT CAG GT
```

Figure S2: Information about the zebrafish *cxcl17* gene (*zgc:158701*).

(**A**) Position and architecture of the zebrafish *cxcl17* gene (*zgc:158701*) in NCBI reference genome (GRCz12tu). The zebrafish *cxcl17* gene (*zgc:158701*) is indicated by a red asterisk. (**B**) Genomic DNA sequence of the zebrafish *cxcl17* gene (*zgc:158701*). (**C**) cDNA sequence (NM_001144821) and encoded amino acid sequence (NP_001138293) of the zebrafish *cxcl17* gene (*zgc:158701*). The primer positions for PCR amplification after reverse transcription were underlined. Two primer pairs were designed for this gene. The information was downloaded from the NCBI gene database (https://www.ncbi.nlm.nih.gov/gene/?term=100151367).

A (Gene position and architecture)

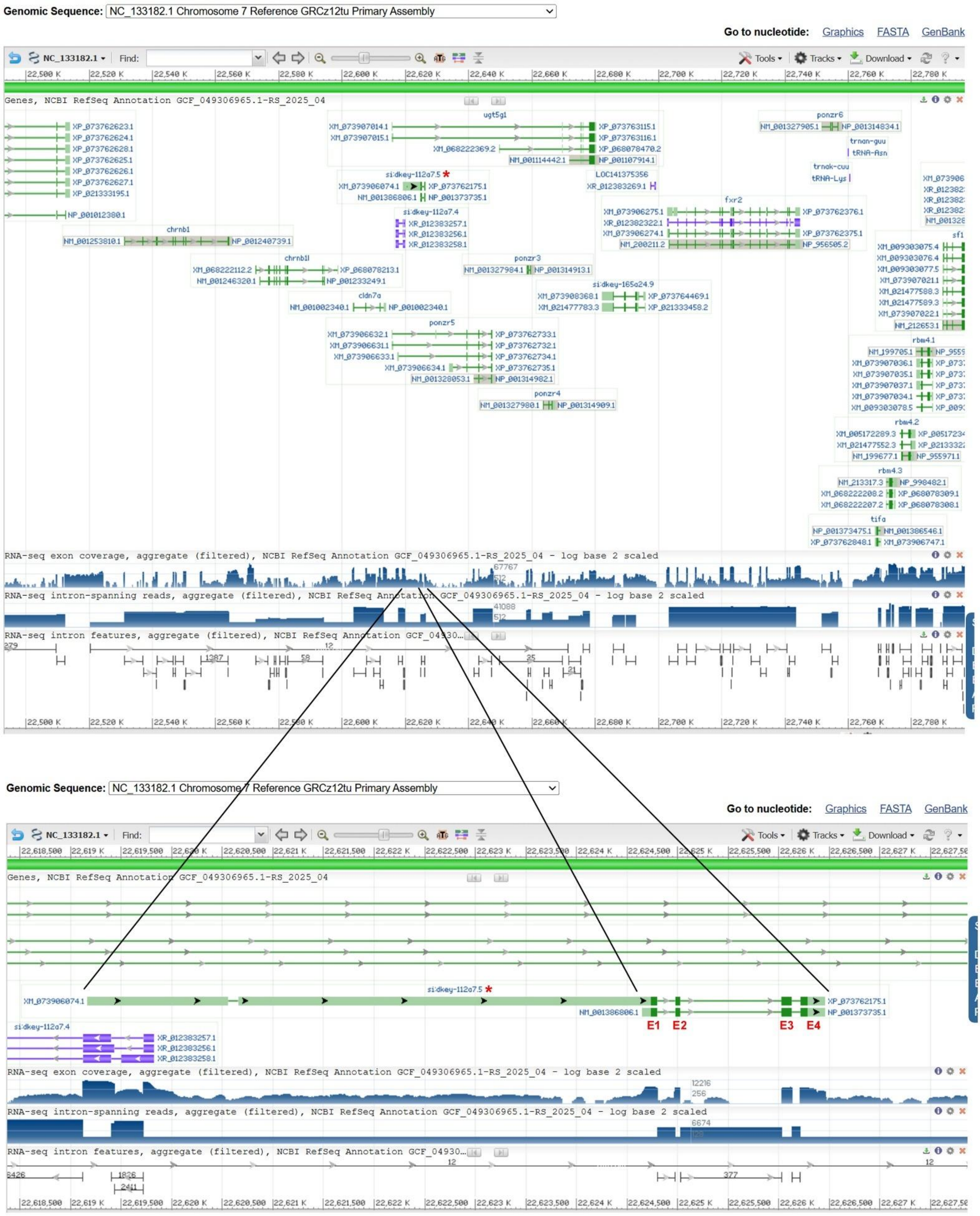

B (genomic DNA sequence, exons in red and highlighted in yellow, coding region underlined)

attgttcaccaaataaatattacaggtcagtgcacttcatcataaggacacaatctattttgctttccttttgacttggactcgcagctaagcacctaatttatttcagatattc
aatcccacacattgtgatctaacaagatcgactttatcagatttaacatgttttctcttgagttttcttgtttagttttgaagtaaacaagaaatataatctttctttttggggt
gaaaatcgtggtatgcaccctgaacatgttgaggcttatttaaaccgttacgcttaaagccctcatgaatggttcagggtataaaagtgggtgaagaggaaagggtgccgatggag
ataatccttgcacgggggataaaaagcaggactcaaatcataccttttcaatccttttggagcagcgatatcgttaagatgaccaaaccaatctgtctggtgtttgccctactga

```
ttctgaccatcattttgtgcaacaactcaggtaattataagcagtggtttacgtcttgtttgcacttactcaagttttgtgtaacatttaatttttctaacatcctaagacaataa
tgtattcatgaaatagaggatgttgtgggttttgatatcaatgtttatgcttgcattgtgagactaatgccacttcttgtcttcacacagtctgttcacagaggagatccatgaaa
caatctgctgtttgtggctgcaaacgtaagaaatccatttgtttttctgtagtttttttcacctaataatgtgtattaaaacactcataaaaataaaggttctttattggcattgag
cattccatgcagagcctttctctacacaaagcatggtaaaaggctatttaaattactgaaacgttcttcgctaagaaaaatggttcttaagaactttaataaaaggttcatgttga
actgttgaaagtggttctacatttttttaataagactgtggtactggtatgctataactacatgtccaggagttcttgttattcattcaagtaaatacaaatgcaaaaagcaaaaaa
acaaaatctgaagtaataaaaaagtttaaccaaaattagaatggcaaaagttagtcgctgagtatgctcaagaaagttccggaaagaggaccaaagcacatttgatatctaatata
aatcataagtaaaccattgaagtatttttccaaaagtaaaagcgtggtcgccatttctcaagtagtctactcctacttaaaatttgtaatataaacaatctttaaagaagtatttg
gtttcaatacaaatgtaagtttataactattttggttatgtagatcatgcatcaaaattatggttagagaaatagaaaaattaagttaaaaaaattgaagtttatctgtaaatttc
attccaacagatataaaaattgagtaagttcaataaggcctagtattgtttcatctgcataataaaatgcaaaaaataataatttttaagtaaatgaagcaataaatttttatcag
tgtgtgtatagtgactgaacgtgagtaactaattgtatacatttttttggccaagtagatgccaaaattcagcacaaatgcttaatggatgctttatatttaagtgtgtgagtgtt
taatttgatgcagatttgagtgtttgatgattgttttgatttgaagcatacgattatttgagattgcctaatgtaataacccaacttttctctttagtttaccctgataagggcct
aaagtgtacaaaaagacccaaccctaaatccagagatgaatactatgaaattctgaagtgcatttgcagggacacccaaatatgtaagtttgaaggattgtgaatgtaaagtaata
ttaccagtgatgaaatataatcattctaataagtcatgtcattcttttgcagtctcaaaaagcagcaggaaagaatatctaaagaggtgcaacaaattttacccttctcttccttt
gtgagctgataaatgtgcaggtttatgaggctcctctgtatcagttaatccatatctataaaaataacacatgcatttattgtctaacgcaactagtggaataataattaaaaaaa
gctctcatttcatcaatgttacatgtgtaattgatctatttgaatgcaataaaggtatgtaaagtgtttttctgttatcactgtgtttgagaaaaagatatgtgttctgtatctca
accggttatctttgtttacagttgcctttatactgatacaggcttctttggctgattttagaccatattttaagggctatgcatgctctaacaagc
```

C (cDNA sequence and encoded amino acid sequence)

```
1       C CGA TGG AGA TAA TCC TTG CAC GGG GGG ATA AAA AGC AGG ACT CAA ATC ATA CCT TTT CAA TCC TTT TGG AGC AGC GAT ATC GTT AAG
        G GCT ACC TCT ATT AGG AAC GTG CCC CCC TAT TTT TCG TCC TGA GTT TAG TAT GGA AAA GTT AGG AAA ACC TCG TCG CTA TAG CAA TTC

89    ATG ACC AAA CCA ATC TGT CTG GTG TTT GCC CTA CTG ATT CTG ACC ATC ATT TTG TGC AAC AAC TCA GTC TGT TCA CAG AGG AGA TCC ATG
      TAC TGG TTT GGT TAG ACA GAC CAC AAA CGG GAT GAC TAA GAC TGG TAG TAA AAC ACG TTG TTG AGT CAG ACA AGT GTC TCC TCT AGG TAC
       M   T   K   P   I   C   L   V   F   A   L   L   I   L   T   I   I   L   C   N   N   S   V   C   S   Q   R   R   S   M

179   AAA CAA TCT GCT GTT TGT GGC TGC AAA CTT TAC CCT GAT AAG GGC CTA AAG TGT ACA AAA AGA CCC AAC CCT AAA TCC AGA GAT GAA TAC
      TTT GTT AGA CGA CAA ACA CCG ACG TTT GAA ATG GGA CTA TTC CCG GAT TTC ACA TGT TTT TCT GGG TTG GGA TTT AGG TCT CTA CTT ATG
       K   Q   S   A   V   C   G   C   K   L   Y   P   D   K   G   L   K   C   T   K   R   P   N   P   K   S   R   D   E   Y

269   TAT GAA ATT CTG AAG TGC ATT TGC AGG GAC ACC CAA ATA TTC TCA AAA AGC AGC AGG AAA GAA TAT CTA AAG AGG TGC AAC AAA TTT TAC
      ATA CTT TAA GAC TTC ACG TAA ACG TCC CTG TGG GTT TAT AAG AGT TTT TCG TCG TCC TTT CTT ATA GAT TTC TCC ACG TTG TTT AAA ATG
       Y   E   I   L   K   C   I   C   R   D   T   Q   I   F   S   K   S   S   R   K   E   Y   L   K   R   C   N   K   F   Y

359   CCT TCT CTT CCT TTG TGA GCT GAT AAA TGT GCA GGT TTA TGA GGC TCC TCT GTA TCA GTT AAT CCA TAT CTA TAA AAA TAA CAC ATG CAT
      GGA AGA GAA GGA AAC ACT CGA CTA TTT ACA CGT CCA AAT ACT CCG AGG AGA CAT AGT CAA TTA GGT ATA GAT ATT TTT ATT GTG TAC GTA
       P   S   L   P   L   *

449   TTA TTG TCT AAC GCA ACT AGT GGA ATA ATA ATT AAA AAA AGC TCT CAT TTC ATC AAT GTT ACA TGT GTA ATT GAT CTA TTT GAA TGC AAT
      AAT AAC AGA TTG CGT TGA TCA CCT TAT TAT TAA TTT TTT TCG AGA GTA AAG TAG TTA CAA TGT ACA CAT TAA CTA GAT AAA CTT ACG TTA

539   AAA GGT ATG TAA AGT G
      TTT CCA TAC ATT TCA C
```

Figure S3: Information about the zebrafish *cxcl17-like* gene (*si:dkey-112a7.5*).

(**A**) Position and architecture of the zebrafish *cxcl17-like* gene (*si:dkey-112a7.5*) in NCBI reference genome (GRCz12tu). The zebrafish *cxcl17-like* gene (*si:dkey-112a7.5*) is indicated by a red asterisk. The long 5'-untranslated region of the so-called transcript XM_073906074 might be due to incorrect annotation. (**B**) Genomic DNA sequence of the zebrafish *cxcl17-like* gene (*si:dkey-112a7.5*). (**C**) cDNA sequence (NM_001386806) and encoded amino acid sequence (NP_001373735) of the zebrafish *cxcl17-like* gene (*si:dkey-112a7.5*). The primers for PCR amplification after reverse transcription were underlined. The information was downloaded from the NCBI gene database (https://www.ncbi.nlm.nih.gov/gene/?term=100536854).

A (primers for PCR amplification)

Dr-CXCL17-F1: 5’ -GTC TCA TTT TGA TTC TGA TTG CCG-3’ ; Dr-CXCL17-R1: 5’ -GT GAC AGT GAA TGC CAA AAG GTC-3’ (pair 1)
Dr-CXCL17-F2: 5’ -ATG AAA ACC ATG AAC TTC CAG ATA-3’ ; Dr-CXCL17-R2: 5’ -TTA TAT TGG CAA ACT GGC TCC TTT-3’ (pair 2)

Dr-CXCL17-like-F: 5’ -TTT TGG AGC AGC GAT ATC GTT AAG-3’ ; Dr-CXCL17-like-R: 5’ -TCA TAA ACC TGC ACA TTT ATC AGC-3’

Dr-GPR25-F: 5’ -A TTG CCC ATC ACT TGT GCA CAG-3’ ; Dr-GPR25-R: 5’ -A AAA AGA CAG ATT GAG AAA CAG ATT-3’

B (PCR products analyzed by agarose gel electrophoresis)

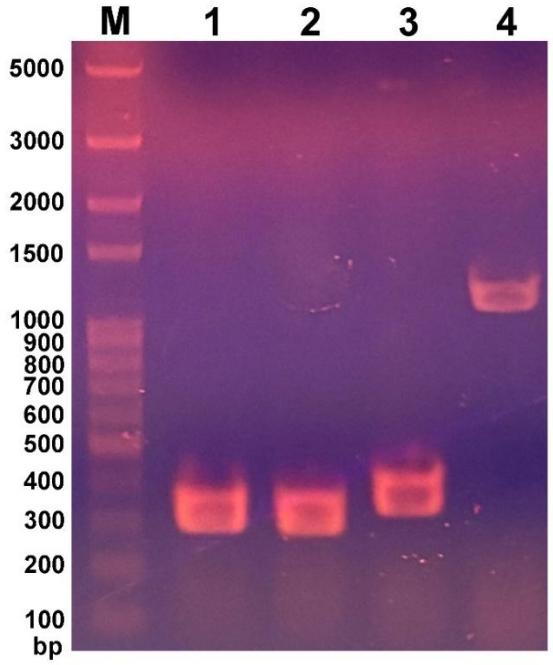

Lane 1: Dr-CXCL17-like
Lane 2: Dr-CXCL17 amplified by pair 2 primers
Lane 3: Dr-CXCL17 amplified by pair 1 primers
Lane 4: Dr-GPR25

Figure S4: cDNA cloning of the zebrafish CXCL17, CXCL17-like, and GPR25.

(**A**) Nucleotide sequence of synthetic oligoes from PCR amplification after reverse transcription. Two primer pairs were used for amplification of Dr-CXCL17. (**B**) Analysis of the PCR products by agarose gel electrophoresis.

6×His-Dr-CXCL17

```
1   CTT TAA GAA GGA GAT ATA ATG CAT CAT CAC CAC CAT CAC CGT CCG CAG GAA GGT AAG TCC GAC AAA TCT GCA GAG
    GAA ATT CTT CCT CTA TAT TAC GTA GTA GTG GTG GTA GTG GCA GGC GTC CTT CCA TTC AGG CTG TTT AGA CGT CTC
                             M   H   H   H   H   H   H   R   P   Q   E   G   K   S   D   K   S   A   E

76  GTT AAA GGC CAT GCT ATG CCT CGC AAA TGC AAC TGT CAA GTG CGT GGT ACT GCG CTG GAT CGC AAC TGT GTG TGT
    CAA TTT CCG GTA CGA TAC GGA GCG TTT ACG TTG ACA GTT CAC GCA CCA TGA CGC GAC CTA GCG TTG ACA CAC ACA
     V   K   G   H   A   M   P   R   K   C   N   C   Q   V   R   G   T   A   L   D   R   N   C   V   C

151 GAA ATG CCA CAC AAA AGC CGT CCG ACC CTC AAT CCA GAA CAG AAA AAC ATG TGC TTA AAG AAG AAA ATT AAA ACC
    CTT TAC GGT GTG TTT TCG GCA GGC TGG GAG TTA GGT CTT GTC TTT TTG TAC ACG AAT TTC TTC TTT TAA TTT TGG
     E   M   P   H   K   S   R   P   T   L   N   P   E   Q   K   N   M   C   L   K   K   K   I   K   T

226 TTT CGC AAA TGC CTG CAG TTT ATG GGT GCA AAC AAG AAA ATC GCG AAA GGC GCC AGT TTG CCG ATT TAA GCG GCC
    AAA GCG TTT ACG GAC GTC AAA TAC CCA CGT TTG TTC TTT TAG CGC TTT CCG CGG TCA AAC GGC TAA ATT CGC CGG
     F   R   K   C   L   Q   F   M   G   A   N   K   K   I   A   K   G   A   S   L   P   I   *

301 GCA CTC GAG CAC CAC
    CGT GAG CTC GTG GTG
```

6×His-Dr-CXCL17-like

```
1   CTT TAA GAA GGA GAT ATA ATG CAT CAT CAC CAC CAT CAC CAG CGT CGC TCC ATG AAA CAA TCT GCG GTG TGT GGC
    GAA ATT CTT CCT CTA TAT TAC GTA GTA GTG GTG GTA GTG GTC GCA GCG AGG TAC TTT GTT AGA CGC CAC ACA CCG
                             M   H   H   H   H   H   H   Q   R   R   S   M   K   Q   S   A   V   C   G

76  TGC AAA CTT TAC CCT GAT AAG GGT CTG AAA TGT ACC AAA CGT CCG AAT CCA AAA TCG CGC GAT GAA TAC TAT GAG
    ACG TTT GAA ATG GGA CTA TTC CCA GAC TTT ACA TGG TTT GCA GGC TTA GGT TTT AGC GCG CTA CTT ATG ATA CTC
     C   K   L   Y   P   D   K   G   L   K   C   T   K   R   P   N   P   K   S   R   D   E   Y   Y   E

151 ATT CTG AAG TGT ATT TGC CGT GAC ACC CAG ATC TTC TCA AAA AGC AGT CGC AAA GAA TAT CTG AAG CGT TGC AAC
    TAA GAC TTC ACA TAA ACG GCA CTG TGG GTC TAG AAG AGT TTT TCG TCA GCG TTT CTT ATA GAC TTC GCA ACG TTG
     I   L   K   C   I   C   R   D   T   Q   I   F   S   K   S   S   R   K   E   Y   L   K   R   C   N

226 AAA TTT TAC CCG TCT CTC CCG TTG TAA GCG GCC GCA CTC GAG CAC CAC
    TTT AAA ATG GGC AGA GAG GGC AAC ATT CGC CGG CGT GAG CTC GTG GTG
     K   F   Y   P   S   L   P   L   *
```

6×His-SmBiT-Dr-CXCL17-like

```
1   CTT TAA GAA GGA GAT ATA ATG CAT CAT CAC CAT CAC CAT GGT GTG ACC GGC TAC CGT CTG TTT GAA GAA ATT CTG
    GAA ATT CTT CCT CTA TAT TAC GTA GTA GTG GTA GTG GTA CCA CAC TGG CCG ATG GCA GAC AAA CTT CTT TAA GAC
                             M   H   H   H   H   H   H   G   V   T   G   Y   R   L   F   E   E   I   L

     G   G   Q   R   R   S   M   K   Q   S   A   V   C   G   C   K   L   Y   P   D   K   G   L   K   C
76  GGC GGC CAG CGT CGC TCC ATG AAA CAA TCT GCG GTG TGT GGC TGC AAA CTT TAC CCT GAT AAG GGT CTG AAA TGT
    CCG CCG GTC GCA GCG AGG TAC TTT GTT AGA CGC CAC ACA CCG ACG TTT GAA ATG GGA CTA TTC CCA GAC TTT ACA

151 ACC AAA CGT CCG AAT CCA AAA TCG CGC GAT GAA TAC TAT GAG ATT CTG AAG TGT ATT TGC CGT GAC ACC CAG ATC
    TGG TTT GCA GGC TTA GGT TTT AGC GCG CTA CTT ATG ATA CTC TAA GAC TTC ACA TAA ACG GCA CTG TGG GTC TAG
     T   K   R   P   N   P   K   S   R   D   E   Y   Y   E   I   L   K   C   I   C   R   D   T   Q   I

226 TTC TCA AAA AGC AGT CGC AAA GAA TAT CTG AAG CGT TGC AAC AAA TTT TAC CCG TCT CTC CCG TTG TAA GCG GCC
    AAG AGT TTT TCG TCA GCG TTT CTT ATA GAC TTC GCA ACG TTG TTT AAA ATG GGC AGA GAG GGC AAC ATT CGC CGG
     F   S   K   S   S   R   K   E   Y   L   K   R   C   N   K   F   Y   P   S   L   P   L   *

301 GCA CTC GAG CAC CAC
    CGT GAG CTC GTG GTG
```

Figure S5: The nucleotide and amino acid sequence of the zebrafish CXCL17 and CXCL17-like overexpressed in *E. coli.*

The amino acid sequence of mature Dr-CXCL17 and Dr-CXCL17-like is shown in red, that of SmBiT in blue.

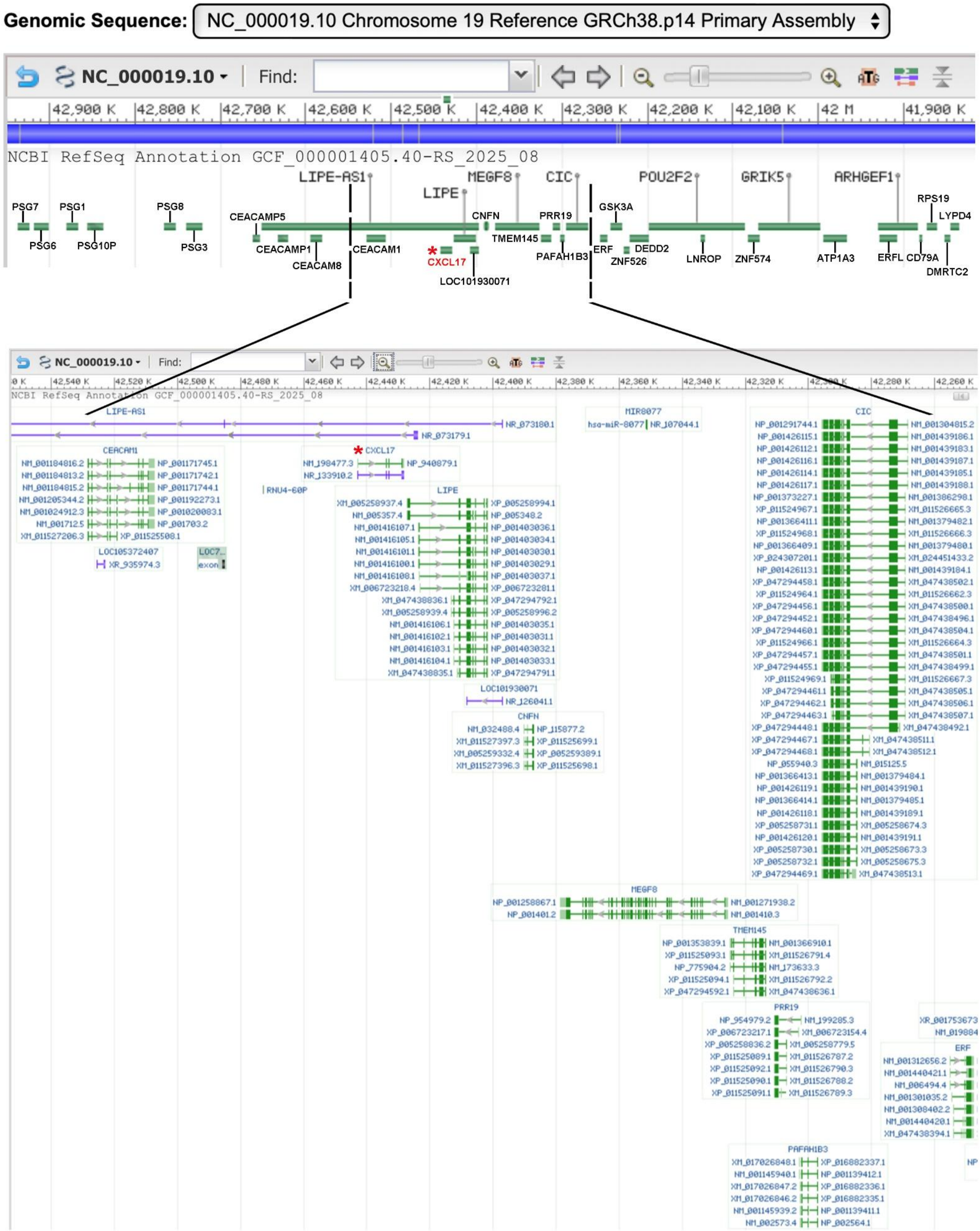

Figure S6: Position of the human *CXCL17* gene in human genome.

The human *CXCL17* gene is indicated by a red asterisk. The information was downloaded from the NCBI gene database (https://www.ncbi.nlm.nih.gov/gene/284340).
